# Identification of cold-inducible microRNAs in grapevine

**DOI:** 10.3389/fpls.2015.00595

**Published:** 2015-08-04

**Authors:** Xiaoming Sun, Gaotao Fan, Lingye Su, Wanjun Wang, Zhenchang Liang, Shaohua Li, Haiping Xin

**Affiliations:** ^1^Key Laboratory of Plant Germplasm Enhancement and Specialty Agriculture, Wuhan Botanical Garden, Chinese Academy of SciencesWuhan, China; ^2^Beijing Key Laboratory of Grape Sciences and Enology, CAS Key Laboratory of Plant Resources, Institute of Botany, Chinese Academy of SciencesBeijing, China; ^3^University of Chinese Academy of SciencesBeijing, China; ^4^Department of Biological Engineering, School of Life Science and Engineering, Southwest Jiaotong UniversityChengdu, China

**Keywords:** *Vitis vinifera*, microRNA, cold-inducible, abiotic stress, Solexa

## Abstract

Low temperature is one of the most important environmental factors that limits the geographical distribution and productivity of grapevine. However, the molecular mechanisms on how grapevine responds to cold stress remains to be elucidated. MicroRNAs (miRNAs) are a class of endogenous small non-coding RNAs that play an essential role during plant development and stress responses. Although miRNAs and their targets have been identified in several *Vitis* species, their participation during cold accumulation in grapevine remains unknown. In this study, two small RNA libraries were generated from micropropagated ‘Muscat Hamburg’ (*V. vinifera*) plantlets under normal and low temperatures (4°C). A total of 163 known miRNAs and 67 putative novel miRNAs were detected from two small RNA libraries by Solexa sequencing. Forty-four cold-inducible miRNAs were identified through differentially expressed miRNAs (DEMs) analysis; among which, 13 belonged to upregulated DEMs while 31 belonged downregulated DEMs. The expression patterns of the 13 DEMs were verified by real-time RT-PCR analysis. The prediction of the target genes for DEMs indicated that miRNA may regulate transcription factors, including AP2, SBP, MYB, bHLH, GRAS, and bZIP under cold stress. The 5′-RLM RACE were conducted to verify the cleavage site of predicted targets. Seven predicted target genes for four known and three novel vvi-miRNAs showed specific cleavage sites corresponding to their miRNA complementary sequences. The expression pattern of these seven target genes revealed negative correlation with the expression level of the corresponding vvi-miRNAs. Our results indicated that a diverse set of miRNAs in *V. vinifera* are cold-inducible and may play an important role in cold stress response.

## Introduction

MicroRNAs (miRNAs) are a class of endogenous small non-coding RNAs (~21 nt in length) that regulate gene expression at the post-transcriptional level by targeting messenger RNAs (mRNA) for cleavage or translational repression (Chen, 2004; Jones-Rhoades et al., 2006; Mallory and Vaucheret, 2006). MiRNAs have been initially identified in *Caenorhabditis elegans* as genes required for temporal control of developmental events (Lee et al., 1993). Since their identification, thousands of miRNAs have been identified in a broad range of mammals, as well as in plants and viruses. The functions of these miRNAs range from maintaining genome stability, developmental patterning, and response to environmental stimuli in defense against viruses and bacteria in eukaryotes (Ramachandran and Chen, 2008).

Given its fundamental roles, plant miRNA was first identified in *Arabidopsis thaliana* by different research groups (Llave et al., 2002; Park et al., 2002; Reinhart et al., 2002) and later in other species. Currently, miRNAs have been reported in 72 plant species; all their sequences have been deposited in the miRBase database (Kozomara and Griffiths-Jones, 2014) (http://www.mirbase.org/). Functional analysis of miRNAs have indicated its critical roles in regulating various metabolism and biological processes, including growth development (Yang et al., 2007; Lelandais-Brière et al., 2010), phytohormone signaling (Liu and Chen, 2009), and flowering and sex determination (Chuck et al., 2009). Furthermore, increasing evidence has shown that plant miRNAs are extensively involved in biotic and abiotic stresses responses (Phillips et al., 2007; Shukla et al., 2008; Katiyar-Agarwal and Jin, 2010; Barrera-Figueroa et al., 2012). For example, miR395 could be induced by sulfate starvation and targets the ATP sulfurylase genes (*APS1, APS3*, and *APS4*) to regulate sulfate metabolism (Jones-Rhoades and Bartel, 2004). Sunkar and Zhu (2004) reported that miR393, miR397b, and miR402 from *Arabidopsis* are upregulated by cold temperature, dehydration, high salinity, and abscisic acid (ABA); whereas miR389a is downregulated under the same stresses. However, miR319c appeared to be only induced by cold temperature (Sunkar and Zhu, 2004). The expression of miR319 in sugarcane is also upregulated during cold stress (Thiebaut et al., 2012). Overexpression of Osa-miR319 could lead to enhanced cold tolerance in rice (Yang et al., 2013).

Grapevine (*Vitis vinifera* L.) is one of the most important and widely cultivated fruit crops in the world; its whole genomic sequence was released in 2007 (Jaillon et al., 2007). Low temperature is one of the important environmental factors that negatively affect grapevine productivity and quality. To address this issue, the genetic mechanisms of cold accumulation in grapevine have been widely studied. However, the miRNA-based regulatory mechanisms under low temperature remain largely unknown. Grapevine miRNAs, including 13 conserved and 5 non-conserved miRNAs, were first isolated from mixed-stage grape berries of *V. vinifera* cv. Nebbiolo (Carra et al., 2009). Many of these miRNAs are differentially expressed in different tissues and during fruit ripening in grapevine (Mica et al., 2010). Pantaleo et al. (2010) found 21 new grapevine-specific miRNAs and 21 other plausible miRNAs in four tissues of *V. vinifera* cv. Pinot Noir (ENTAV115). Many of the previously known miRNAs showed tissue-specific expression patterns. Wang et al. (2011a,b, 2012) also identified miRNAs in different tissues (young leaves, large leaves, stems, tendrils, inflorescences, flowers, and young berries) of *V. vinifera* cv. Summer Black and *V. amurensis*, and predicted their target genes to investigate their functions. To date, 186 grapevine miRNAs have been deposited into the miRBase 21 (June 2014) (Kozomara and Griffiths-Jones, 2014).

In this study, we report the identification of known and putative novel miRNAs that may participate in cold signal transduction in grapevine. Two small RNA libraries were generated from normal and low temperature-treated micropropagated plantlets of ‘Muscat Hamburg’ (*V. vinifera*). Based on deep sequencing technology and published grape genome sequences (Jaillon et al., 2007), a whole-genome-wide identification of cold-stress related miRNAs was performed. Real-time RT-PCR was used to verify the expression pattern of cold-inducible miRNAs and its putative targets, which would help illustrate the roles of miRNAs during cold stress response in grapevine.

## Materials and methods

### Plant materials

Micropropagated ‘Muscat Hamburg’ (*V. vinifera*) plantlets were grown on 1/2 B5 medium with 30 g L^−1^ of sucrose in a growth chamber at 26°C. The average photosynthetic photon flux (PPF) was 100 μmol m^−2^ s^−1^ with a 16 h light and 8 h dark cycle. At the age of 6 weeks, plantlets used for cold treatment were transferred to another growth chamber under the same conditions, except for temperature (4°C). The shoot apex with one well developed leaf was collected at 0, 2, 4, 8, 24, and 48 h after cold treatment. Three plantlets per condition and time points were pooled together; for next generation sequencing analysis only 1 replicate and 2 time point (0 and 4 h) were considered, while for real time PCR analysis, 3 biological replicates (for a total of 9 plantlets) and 6 time points were sampled. After the collection, all samples were immediately frozen in liquid nitrogen and stored at −70°C.

### Small RNA libraries construction and Solexa sequencing

Total RNA was extracted from collected samples using Column Plant RNAOUT 2.0 kit (Tiandz, Beijing, China), following the instructions of the manufacturer. RNA concentration and quality were detected using Agilent 2100 Bioanalyzer. Two small RNA libraries of *V. vinifera* were generated and sequenced using Illumina Hiseq 2000 with 50 bp read length. Multiplexing was applied during sequencing. Libraries were constructed according to the TruSeq® Small RNA Sample Preparation Guide kit (Illumina, USA). Briefly, total RNA was purified by electrophoretic separation on a 15% TBE urea denaturing polyacrylamide gel electrophoresis (PAGE) gel and small RNA regions corresponding to the 18–30 nt bands in the marker lane were excised and recovered. Then the 18–30 nt small RNAs were ligated to a 5′-adaptor and a 3′-adaptor sequentially by T4 RNA ligase. The adapter-ligated small RNAs were subsequently transcribed into cDNA by SuperScript III Reverse Transcriptase (Invitrogen, USA) and then PCR-amplified for 17 cycles using the adaptor primers. The PCR products were purified for high-throughput sequencing. The sequencing was performed by Beijing Genomics Institute (BGI, Shenzhen, Guangdong, China). All small RNA sequence data generated by deep sequencing of the two small libraries have been deposited in the Gene Expression Omnibus (GEO) database under the accession number GSE68970.

### Identification of known and novel miRNAs

Clean reads were obtained by discarding read lengths shorter than 18 nt or longer than 30 nt, while removing the adaptors, low quality tags, and contaminants from the raw reads. The read length distributions of unique and total reads were summarized. Small RNA tags were mapped to the *V. vinifera* cv. Pinot Noir (PN40024) genomes using the SOAP2 program. The program was performed using the following parameters: soap -v 0 -r 2 -M 0 -a clean.fa -D ref_genome.fa.index -o match_genome.soap. The matched sequences were then queried against snRNAs, snoRNAs, scRNAs, rRNAs, and tRNAs from NCBI Genbank database (http://www.ncbi.nlm.nih.gov/blast/Blast.cgi) and Rfam database (http://www.sanger.ac.uk/Software/Rfam) by Blastn search with the parameters, blastall -p blastn -F F -e 0.01. Any small RNAs having exact matches to these sequences were excluded from further analyses. The sequences of precursor miRNAs were downloaded from the miRBase 21 (http://www.mirbase.org/) and aligned with unique miRNA sequences using Blastn, with the parameters, blastall-p blastn-F F -e 0.01. Sequences were considered as known miRNAs in *V. vinifera* with a maximum of two mismatches. No abundance filters were set during known miRNA screening. The secondary structures of the obtained miRNA sequences were confirmed using the online software mfold (http://www.bioinfo.rpi.edu/applications/mfold/). The remaining unknown sRNAs were analyzed, and novel miRNAs were predicted using Mireap (https://sourceforge.net/projects/mireap/). The novel miRNAs were identified according to the previously reported criteria (Ambros et al., 2003; Meyers et al., 2008). Breifly, a novel miRNA would be reported if the following criteria were met: (1) miRNA sequence length is 18–25; (2) miRNA reference sequence length is 20–23; (3) Maximal copy number of miRNAs on reference is 20; (4) Maximal free energy allowed for a miRNA precursor is -18 kcal/mol; (5) Maximal space between miRNA and miRNA^*^ is 30 nts; (6) Minimal base pairs of miRNA and miRNA^*^ is 16; (7) Maximal bulge of miRNA and miRNA^*^ is 4; (8) Maximal asymmetry of miRNA/miRNA^*^ duplex is 4; (9) Flank sequence length of miRNA precursor is 20 nts; (10) More than 50 reads are mapping on the precursor in one of the two libraries.

### Differentially expressed miRNAs analysis

Differently expressed miRNAs (DEMs) were calculated according to our previous publication (Xin et al., 2013). Transcripts per million (TPM) reads were used to evaluate the relative expression levels of each miRNA in two small RNA libraries. If the read count of a given miRNA was zero, the TPM was modified to 0.01 for further analysis; if the miRNA TPM of two libraries was < 10, on account of low expression, the miRNA was neglected in the analysis of differential expression. The fold-change between the CT and NCT libraries was calculated using the following equation: Fold-change = log_2_ (CT/NCT). The miRNAs with fold-changes >1 or <-1 and *p-values* ≤0.001 were considered upregulated or downregulated in response to cold stress, respectively. The *p-value* was calculated according to the previously established methods (Man et al., 2000).

### Identification and functional annotation of the target genes

Target predictions were performed based on the criteria described by Allen et al. (2007) and Schwab et al. (2005), using the psRNATarget server (http://plantgrn.noble.org/psRNATarget/) (Dai and Zhao, 2011) and default parameters. Published researches (Baek et al., 2008; Reczko et al., 2012; Hausser et al., 2013) revealed that miRNAs were able to bind both the coding sequence (CDs) and the untranslated regions (UTRs) of a transcript, regulating the expressions of the targets. We scanned the *V. vinifera* transcript library released by the Wine grape genomic sequencing project (http://genomes.cribi.unipd.it/DATA/) to identify the putative homolog genes and the functions of the potential targets. GO analysis was conducted using the AgriGO database (http://bioinfo.cau.edu.cn/agriGO/) (Du et al., 2010); the result is shown in **Figure 3**.

### Detection and analysis the expression pattern of cold-inducible miRNAs

Total RNA was extracted from grape plantlet leaves subjected to cold stress at 4°C for 0, 2, 4, 8, 24, and 48 h using the Column Plant RNAOUT 2.0 kit (Tiandz, Beijing, China). RNase-free DNase I (Promega, USA) was used to degrade any DNA present in the extracted RNA. Poly(A)-tailed real-time RT-PCR was performed using the SYBR PrimeScript miRNA RT-PCR Kit (TaKaRa, Dalian, China), according to the instructions of the manufacturer. The miRNA PrimeScript RT Enzyme Mix [poly(A) polymerase and PrimeScript RTase included] was used in adding poly(A)-tailed and reverse transcription using a universal adapter primer (oligo-dT and uni-miR qPCR primer binding sits included). Approximately 1 μg of total RNA was used in a 20 μL reaction system. The mix was incubated at 37°C for 60 min, then at 85°C for 30 s.

Amplification of the miRNAs with poly(A) tails was performed using gene-specific primers (Table S8) and the uni-miR qPCR primer, which was included in the kit. Poly (A)-tailed real-time RT-PCR was carried out in an ABI SteopOneplus™ Real-Time PCR system (Applied Biosystems) using FastStart Universal SYBR Green Master (Roche, Shanghai, China). Each reaction was replicated three times for each biological sample, using a total of three biological replicates. The 5S rRNA was used as an internal control (Wang et al., 2011b), and the sample without template was used as a negative control. The Ct values and real-time PCR efficiencies were obtained using LinRegPCR version 2015.1 (Ruijter et al., 2009), and the relative expression and standard errors for each sample were calculated using Biogazelle qbasePLUS (Hellemans et al., 2007).

### Verification of the miRNAs cleavage site by 5′ RLM-RACE

Total RNA was isolated from cold-treated shoot apex for 4 h, using the approach mentioned above. The 5′ RLM-RACE was performed using GeneRacer™ Kit (Invitrogen, USA), according to the instructions of the manufacturer. Gene-specific reverse primers and gene-specific reverse nested primers were designed from the predicted targets and used in combination with the 5′ adapter primers (GeneRacer™ 5′ Primer, GeneRacer™ 5′ Nested Primer) to amplify the cleaved transcripts. The PCR products were detected by agarose gel electrophoresis. The target fragments were cloned into pGEM-T Easy vectors (Promega, USA) and sequenced using the universal primers SP6 and T7.

### Expression pattern analysis of target genes

Total RNA was extracted from grape plantlet leaves subjected to cold stress at 4°C for 0, 2, 4, 8, 24, and 48 h. DNA was digested as the method mentioned above. cDNA was synthesized using the SuperScript III Reverse Transcriptase (Invitrogen, USA) with Oligo-dT, according to the instructions of the manufacturer. The approach of the real-time RT-PCR step and the data analysis were the same as previously performed. The malate dehydrogenase gene (MDH, GenBank accession number: EC921711) and β-actin (GenBank accession number: EC969944) were used as the internal controls (Wang et al., 2014b). Gene-specific primer pairs were designed using Primer Premier 5 around the cleavage site of the target genes, the sense primers were at the upstream of the cleavge site and the antisense primers were at the downstream. All the primer sequences are shown in Table S11. The expression correlation between miRNA and its target gene was calculated by Pearson correlation coefficient.

## Results

### Small RNA library construction and sequencing

**Figure 1:**
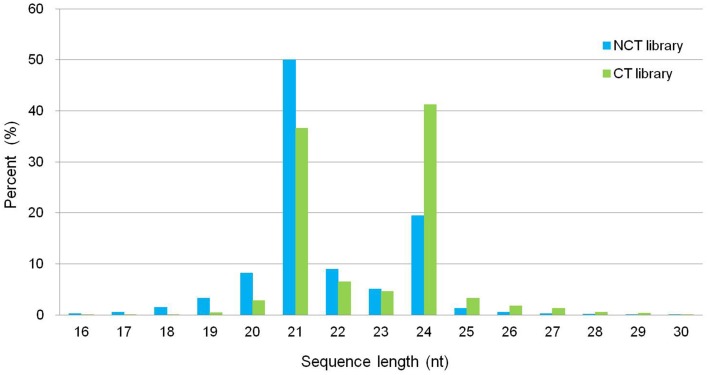
**Length distribution and abundance of small RNAs in the NCT and CT library from micropropagated ‘Muscat Hamburg’ plantlets**. NCT library: library constructed using plantlets grown in growth chamber at 25°C without any stress; CT library: library constructed using plantlets treated at 4°C for 4 h.

We constructed two small RNA libraries, namely, non-cold-treated (NCT) and cold treated (CT), from micropropagated plantlets of ‘Muscat Hamburg’ to identify cold-inducible miRNAs in *V. vinifera*. The NCT small RNA library was constructed using the plantlets grown at 25°C, whereas the CT small RNA library was constructed with micropropagated plantlets subjected to 4°C for 4 h to observe the change in miRNAs in the early stage during cold stress. After sequencing, 21,749,505 and 26,320,458 reads were generated for NCT and CT libraries, respectively (Table S1). The size distribution of all unique small RNAs is summarized in Figure 1. The displayed length of small RNA sequence ranged from 16 to 30 nt; the two major size classes were 21 nt (50.07% in NCT and 36.63% in CT library) and 24 nt (19.43% in NCT and 41.21% in CT library). The abundance of small RNAs from 16 to 23 nt in the NCT library was higher than that in the CT library. However, 24 to 30 nt small RNAs were less abundant in the NCT library than in the CT one (Figure 1).

Based on a comparison with 12 × *V. vinifera* cv. Pinot Noir (PN40024) (Jaillon et al., 2007) and related bioinformatics analysis, there were 57.54 and 59.46% unique sRNAs mapping to the genome in the NCT and CT library, respectively (Table S2). These small RNAs were divided into the following categories: rRNA, snRNA, snoRNA, tRNA, exon_sense, exon_antisense, intron_sense, intron_antisense, repeat associate small RNA, unannotated small RNA, and miRNA candidate sequences. The candidate miRNA sequences represented only 0.03% NCT and 0.05% CT of the unique small RNAs (Table S3). Most sequences (62.33% of the total unique sequences in the NCT library and 66.28% in the CT library) were unannotated (Table S3). A total of 6,404,541 candidate miRNA sequences in the NCT library and 6,148,881 in the CT library were identified, which finally comprised 1431 and 1479 unique sequences, respectively (Table S3).

### Identification of known miRNAs

The candidate miRNA sequences were compared with published miRNAs from other plant species by Blastn search against miRbase 21 (June 2014) to identify the known miRNAs in *V. vinifera*. We identified 163 known miRNAs, belonging to 22 conserved miRNA families and 24 non-conserved miRNA families (Table 1 and Table S4). All conserved miRNA families were detected in our study. In most cases, multiple members from one known miRNA family are present, with significant divergence among them. The miR169 family was the largest identified family, with 23 members distinguished by internal nucleotide diversity. The medium miRNA families were miR395, miR156, miR166, and miR399, with 13, 9, 8, and 8 members, respectively. Among the remaining 40 miRNA families, 28 contained two to seven members and 12 contained a single member (Table S4). Besides, many sequence-variants were identified for almost each member of the known miRNAs (Table S5). Only annotated mature sequence was considered for the further analysis.

**Table 1:** **Known miRNA families identified in ‘Muscat Hamburg’ (***V. vinifera***) leaves from the plantlets under non-cold-treated (NCT) condition and subjected to cold stress (CT) at 4°C**.

**miRNA families**	**NCT reads**	**CT reads**
**CONSERVED miRNA FAMILIES**		
**miR156**	**11426364**	**9449021**
**miR159**	**8151**	**8511**
**miR160**	**241**	**148**
**miR162**	**3519**	**4297**
**miR164**	**99307**	**194057**
**miR166**	**5931116**	**6634940**
**miR167**	**1112300**	**1141665**
**miR168**	**93654**	**124041**
**miR169**	**19528**	**13759**
**miR171**	**2849**	**1785**
**miR172**	**1180**	**651**
**miR319**	**106**	**74**
**miR390**	**9616**	**5849**
**miR393**	**2**	**6**
**miR394**	**58**	**47**
**miR395**	**76777**	**15625**
**miR396**	**3869**	**5126**
**miR397**	**4457**	**2187**
**miR398**	**512**	**244**
**miR399**	**124**	**259**
**miR403**	**1049**	**767**
**miR408**	**5085**	**4116**
**NON-CONSERVED miRNA FAMILIES**		
**miR2111**	**426**	**875**
**miR2950**	**315**	**266**
**miR3623**	**14101**	**15903**
**miR3624**	**1823**	**6328**
**miR3625**	**197**	**157**
**miR3626**	**39**	**50**
**miR3629**	**173**	**249**
**miR3630**	**87**	**41**
**miR3631**	**144**	**192**
**miR3632**	**165**	**147**
**miR3633**	**10427**	**6739**
**miR3634**	**377**	**863**
**miR3635**	**551**	**1124**
**miR3636**	**8861**	**28264**
**miR3637**	**245**	**675**
**miR3638**	**2**	**6**
**miR3639**	**2**	**7**
**miR3640**	**641**	**3495**
**miR447**	**45**	**16**
**miR477**	**48**	**16**
**miR479**	**8012**	**7961**
**miR482**	**482**	**481**
**miR535**	**2199273**	**2898719**
**miR828**	**6**	**0**

*The number of each miRNA family in the table was raw data. Detailed information of each miRNA member was shown in Table S4*.

Read numbers based on expression level analysis showed significant differences in known miRNA families, which ranged from only one to more than ten million reads. The most abundant miRNA family was miR156, with 11,426,364 and 9,449,021 reads in the NCT and CT libraries, respectively (Table 1). Nine miRNA families (miR164, miR166, miR167, miR168, miR169, miR395, miR3623, miR3633, and miR3636) were strongly expressed, with more than 10,000 reads detected in at least one of the two libraries. Eleven other miRNA members showed moderate expression level, ranging from 1000 to 10,000 reads in each library (Table 1).

### Identification of putative novel miRNAs

We used previously reported criteria to predict putative novel miRNAs in ‘Muscat Hamburg’ grape (Ambros et al., 2003; Meyers et al., 2008). The sequences less than 50 reads in both CT and NCT libraries were neglected. After screening, a total of 67 novel miRNAs were identified from the two libraries (Table S6). The precursor of each novel miRNA could form a proper secondary hairpin structure with a maximal free energy (MFE) of −18 kcal/mol (Figure S1). The MFE of the identified novel miRNAs ranged from −18.54 kcal/mol to −135.24 kcal/mol. These putative miRNA sequences were compared with all nucleotide sequences in the NCBI databases by Blastn search. The result showed that no homolog exists in any other plant species, suggesting that these newly identified candidate miRNAs are all *Vitis*-specific.

Compared with conserved miRNAs, the expression levels of the majority of putative novel miRNAs were relatively low. However, some putative novel miRNAs were abundant; for instance, novel_mir_5, novel_mir_20, and novel_mir_25 exceeded 10,000 reads in the NCT and CT libraries) (Table 2 and Table S6). Many putative novel miRNAs were detected only in one library and a few of them were detected with a relatively high abundance. As shown in Table 2, novel_mir_4 and novel_mir_6 were detected only in the NCT library, with 3445 and 4288 counts, respectively, whereas novel_mir_39 and novel_mir_42 were only present in the CT library, with 3916 and 2897 counts, respectively.

**Table 2:** **High abundant novel miRNAs identified in ‘Muscat Hamburg’ (***V. vinifera***) leaves from the plantlets under non-cold-treated (NCT) condition and subjected to cold stress (CT) at 4°C**.

**Novel miRNA**	**Novel miRNA sequence**	**Length (nt)**	**NCT reads**	**CT reads**	**Location**	**MFE (kcal/mol)**
novel_mir_5	CAUGGGCGGUUUGGUAAGAGG	21	25311	23311	chr1:3865565:3865681:+	−46.2
novel_mir_25	UCGCAGGAGAGAUGACGCCGU	21	10867	20765	chr6:3976407:3976496: −	−48.4
novel_mir_20	AGAAGAGAGAGAGUACAGCUA	21	14210	11480	chr5:19124470:19124728: −	−65.6
novel_mir_64	UCCCAGGAGAGAUGGCACCUGC	22	4690	8651	chr17:5928104:5928188:+	−43
novel_mir_32	UGACAAAGAGAGAGAGCACAC	21	9743	8339	chr7_random:1422270:1422383: −	−52.2
novel_mir_33	CCGAGGGAGAGAGCGAGAGGA	21	2611	3074	chr8:14593879:14594080: −	−98.9
novel_mir_65	GUCAACCAGCAACUCUCGCGG	21	341	1357	chr17:2693545:2693757: −	−115.3
novel_mir_55	CUAGAGAUUGUGGAUUAGGCU	21	1844	1294	chr14:10959011:10959186:+	−20.55
novel_mir_30	UUCUCGGACCAGGCUUCAUUC	21	1910	1156	chr7:19450174:19450334:+	−64.3
novel_mir_57	UCUGAACUCUCUCCCUCAUGGC	22	973	851	chr14:24560621:24560731: −	−54.7
novel_mir_7	UGGGGUACGAACUAGAGGUGG	21	903	847	chr1:7530676:7530798:+	−30.26
novel_mir_37	UUCCUCAAGUAGACAUGCAUG	21	969	773	chr9_random:179159:179348:+	−36.71
novel_mir_13	UGCCAAGAAGCACAUUCCUCC	21	1195	661	chr3:17003551:17003669: −	−81.3
novel_mir_60	ACGGAGUGGAAGAGGGGAGGAG	22	370	654	chr16:19451213:19451502:+	−75.96
novel_mir_36	UUGCUGAGAGAGUCGUCUGCC	21	554	639	chr9:19105535:19105615: −	−38.5
novel_mir_21	UUAGAUGAUCAUCAACAAACA	21	479	499	chr5:24742118:24742235: −	−45.5
novel_mir_53	UGGAGAAGGGGAGCACGUGCA	21	355	471	chr14:1414567:1414684:+	−59
novel_mir_47	UGUGGAUGAGAAGAGAUGCGA	21	330	381	chr10:16107042:16107232:+	−72
novel_mir_10	UGAAGAGGUGGAGAGUGGAGUG	22	259	313	chr11:16101897:16102055:+	−85.3
novel_mir_29	UGGAAGCAAUCAGGAGACUUG	21	343	130	chr7:17201643:17201884:+	−105.72
novel_mir_6	UUCCCAAGACCCCCCAUGCCAA	22	4288	0	chr1:3865822:3865924:+	−58.1
novel_mir_4	UUUCCACGGCUUUCUUGAACU	21	3445	0	chr1:1997808:1997989:+	−57.1
novel_mir_27	UUAUGGAUGGCAGAAGGUUUA	21	1625	0	chr6:13594470:13594631: −	−33.1
novel_mir_39	AAUGGGCUGAUUGGGAUAAAA	21	0	3916	chr14:19755471:19755581: −	−53.9
novel_mir_42	UUCCACGGCUUUCUUGAACUU	21	0	2897	chr1:1997803:1997999:+	−61.3
novel_mir_45	AGAUGAUGUAUGGAAUGAAAUU	22	0	521	chr8:22256980:22257050:+	−19.9
novel_mir_44	AAGCAAUCAGGAGACUUGUAG	21	0	342	chr7:17201646:17201881:+	−100.42

*Novel miRNAs shown in the table were detected more than 300 reads in one of the two libraries. Novel miRNA is the name of detected novel miRNAs. Genomic locations of pre-miRNA are listed. +, sense strand; −, anti-sense strand. MFE means the maximal free energy contained in the secondary hairpin structure of each novel miRNA precursor*.

### Identification of differentially expressed miRNAs (DEMs) during the early period of cold stress

The expression level of each mature miRNA was normalized to transcripts per million (TPM) and compared between the NCT and CT library to identify the cold-inducible miRNAs in grapevine. The details of DEMs, including original TPM, fold-change, and P value, are shown in Table S7. To increase the robustness of the data, only the miRNAs with more than 10 TPM in at least one of the library were considered. The miRNAs with at least 2-fold difference in expression during cold treatment are displayed in Table 3. A total of 11 upregulated miRNAs (including 4 novel miRNAs detected only in the CT library) and 33 downregulated miRNAs (including 4 novel miRNAs detected only in the NCT library) were identified during the cold treatment. Among these DEMs, there were 14 unique sequences. Many of DEMs were identical sequences, such as vvi-miR156b-d, vvi-miR171acdi, vvi-miR395a-m, and vvi-miR398bc. The precursors of these miRNAs showed high sequence similarity with its family members.

**Table 3:** **List of differentially expressed miRNAs (DEMs) from the plantlet leaves under non-cold-treated (NCT) condition and subjected to cold stress (CT) at 4°C in ***V. vinifera*****.

**miRNA name**	**NCT-TPM**	**CT-TPM**	**Fold-change (log2^CT∕NCT^)**	***P*-value**
vvi-miR3640^*^	2.76	51.63	4.22	2.22E-265
vvi-miR3640	27.25	83.23	1.61	9.18E-151
vvi-miR3624	85.36	244.18	1.52	0
vvi-miR3636	414.93	1090.49	1.39	0
vvi-miR3637^*^	7.5859	18.0585	1.25	7.30E-24
vvi-miR3634	14.56	31.80	1.13	7.97E-35
novel_mir_65	15.97	52.36	1.71	4.33E-104
novel_mir_44	0.01	13.20	10.37	5.85E-90
novel_mir_45	0.01	20.10	10.97	1.10E-136
novel_mir_42	0.01	111.79	13.45	0
novel_mir_39	0.01	151.10	13.88	0
vvi-miR171a/i	24.44	12.04	−1.02	6.02E-24
vvi-miR171c/d	27.96	13.74	−1.03	2.71E-27
vvi-miR156d	11590.51	5547.77	−1.06	0
vvi-miR156b	11569.63	5527.86	−1.07	0
vvi-miR156c	11422.59	5431.93	−1.07	0
vvi-miR172d	55.02	24.77	−1.15	1.41E-62
vvi-miR397a	208.71	84.39	−1.31	4.20E-283
vvi-miR3633b^*^	13.25	5.29	−1.33	5.23E-20
vvi-miR398b/c	11.99	4.67	−1.36	6.36E-19
vvi-miR3633b	10.68	3.47	−1.62	1.18E-21
vvi-miR395a-m	276.56	46.38	−2.58	0
novel_mir_29	16.06	5.02	−1.68	2.29E-33
novel_mir_13	55.96	25.51	−1.13	4.42E-62
novel_mir_30	89.44	44.61	−1.00	1.09E-80
novel_mir_6	200.79	0.01	−14.29	0
novel_mir_4	161.32	0.01	−13.98	0
novel_mir_27	76.09	0.01	−13.89	0
novel_mir_24	10.21	0.01	−10.00	5.33E-76

*MiRNA star denotes the small RNA processed from the hairpin arm opposite the mature miRNA*.

### Validate the DEMs by real-time RT-PCR analysis

**Figure 2:**
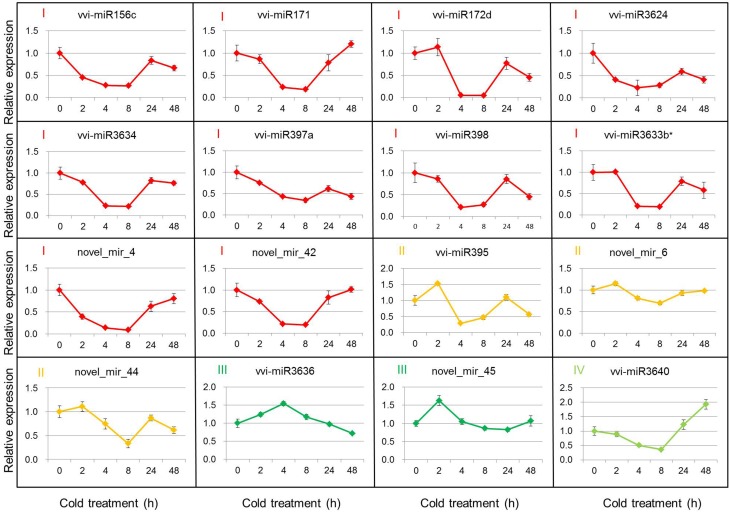
**Real-time RT-PCR validations of the cold-inducible vvi-miRNAs**. Total RNA was extracted from grape leaves from micropropagated plantlets subjected to low temperature at 4°C for 0, 2, 4, 8, 24, and 48 h. The level of expression was normalized to that of 5S rRNA. The normalized vvi-miRNA levels in 0 h were arbitrarily set to 1. Error bars represent the standard deviations of three PCR replicates of a single reverse transcription reaction.

Based on the DEMs analysis in the two libraries, 11 known miRNAs and 5 putative novel miRNAs were selected to verify their expression patterns through poly(A)-tailed real-time RT-PCR. The real-time RT-PCR results show the expression profiles of miRNAs at 4°C for 0, 2, 4, 8, 24, and 48 h after cold initiation (Figure 2). The expression of 13 miRNAs showed the same variation trend at 4 h after cold stress between the real-time RT-PCR and sequencing results, whereas the expression patterns of three miRNAs (vvi-miR3624, vvi-miR3634, and vvi-miR3640) were increased during cold treatment by sequencing, but opposite results were detected from the real-time RT-PCR analysis. In order to prove the reliability of these results, 2 non-differentially expressed miRNAs (vvi-miR3623 and novel_mir_5) were also verified as a control. The results showed the expression of both vvi-miR3623 and novel_mir_5 was no significant changes during the cold treatment and had the same variation trend at 4 h after cold stress with the sequencing results (Figure S2).

The 16 selected DEMs were divided into four groups, according to their expression patterns (Figure 2). Group I included 10 miRNAs (vvi-miR156c, vvi-miR171, vvi-miR172d, vvi-miR397a, vvi-miR398, vvi-miR3624, vvi-miR3633b^*^, vvi-miR3634, novel_mir_4, and novel_mir_42), and their expression decreased rapidly and reached the minimum level at 4 or 8 h, then increased gradually. The expression of Group II miRNAs (vvi-miR395, novel_mir_6, and novel_mir_44) increased at 2 h and decreased at 8 h after the plantlets were subjected to cold stress. The expressions of Groups I and II miRNAs were downregulated in 4°C cold stress. During the cold stress, the average expression level of these miRNAs was lower than the control (0 h). Vvi-miR3636 and novel_mir_45 were categorized into Group III. Their expressions first increased and reached the maximum level, then decreased. Group IV had only one miRNA (vvi-miR3640), and its expression was downregulated until 8 h after cold stress initiation, but increased by up to 2-fold at 48 h. We can conclude that the expressions of Groups III and IV were upregulated around 4 h and after 24 h cold stress, respectively. The expression profile analysis by real-time RT-PCR confirmed the existence of these miRNAs in *V. vinifera*, and suggests that the previous miRNAs may be involved in cold stress responses.

### Target gene prediction for cold-inducible miRNAs

For the 44 DEMs, the target genes were predicted in the psRNATarget database to illustrate the putative mRNAs regulated by miRNAs under cold stress. The rules used for target prediction were based on Allen et al. (2007) and Schwab et al. (2005). The detailed annotation results of the prediction are shown in the Table S8. Among these 44 miRNAs, only novel_mir_65 and novel_mir_24 had no predicted targets. A total of 147 target genes were predicted from the rest of the 42 cold-inducible miRNAs. Most of the miRNAs had multiple distinct targets; however, vvi-miR3633b^*^ and novel_mir_30 targeted only one mRNA.

**Figure 3:**
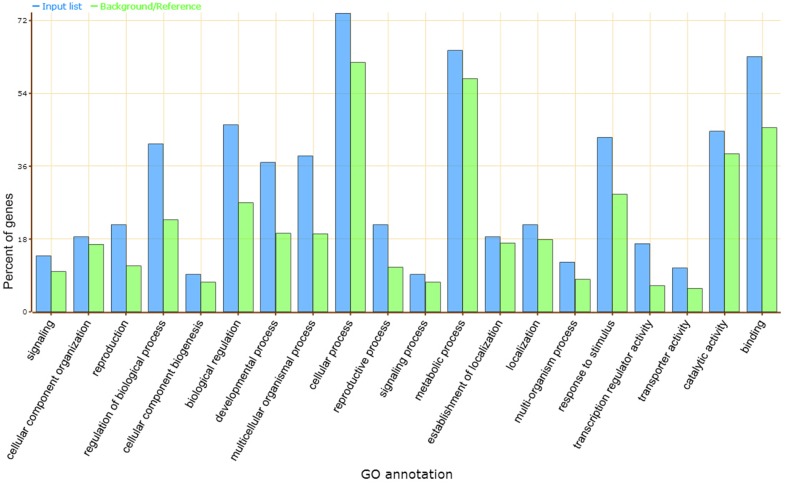
**GO analysis of vvi-miRNA target genes**. Blue and green bars indicate the enrichment of miRNA target genes in GO terms and the percentage of total annotated grapevine genes mapping to GO terms, respectively.

Gene ontology (GO) analysis showed that the predicted target genes may be involved in a broad range of biological processes, such as cellular process, metabolic process, biological regulation, catalytic activity, and response to stimulus (Figure 3). Most of these processes were classified as transcription factors and functional proteins in plant metabolism and abiotic stress responses. Several transcription factors were found in the target genes, including the squamosa promoter binding (SBP) protein (the putative target of vvi-miR156), APETALA2 (AP2, vvi-miR172), basic helix-loop-helix (bHLH, vvi-miR3640^*^), GRAS (novel_mir_39), bZIP (novel_mir_4, novel_mir_42), and MYB-like binding protein (novel_mir_13). Other predicted target genes included proteins such as multicopper oxidase for vvi-miR397a, novel_mir_29, and novel_mir_44; sulfate transporter protein for vvi-miR395; E3 ubiquitin ligase for vvi-miR3634; WD-40 repeat protein for vvi-miR172d; and sugar transporter protein for vvi-miR3630.

### Cleavage sites and expression pattern analysis of seven predicted target genes

**Figure 4:**
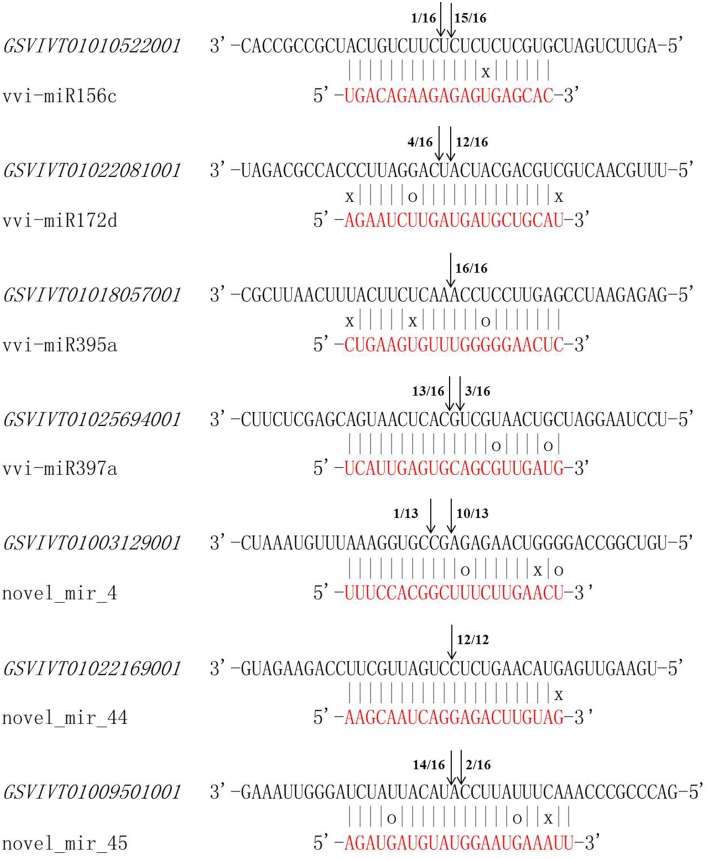
**Mapping of target gene cleavage sites by 5′ RLM-RACE**. For each vvi-miRNA, the partial target sequence (black) is shown at the top and the vvi-miRNA sequence at the bottom (red). Watson-Crick pairing (vertical dashes), G:U wobble pairing (O) and mismatched bases pairing (X) are indicated. Arrows indicate cleavage sites. Numbers indicate the fraction of cloned PCR products terminating at different positions.

The 5′ RLM-RACE experiment was carried out to identify the cleavage sites of the predicted vvi-miRNA target genes. The targets genes of the four known (vvi-miR156c, vvi-miR172d, vvi-miR395a, and vvi-miR397a) and three novel DEMs (novel_mir_4, novel_mir_44, novel_mir_45) were used for the following analysis. The seven target genes were *GSVIVT010 10522001, GSVIVT01022081001, GSVIVT01018057001, GSVIVT01025694001, GSVIVT010 03129001, GSVIVT01022169001*, and *GSVIVT01009501001*. All seven predicted target genes had specific cleavage sites that correspond to the miRNA complementary sequences (Figure 4). The target cleavage sites were concentrated at around the 10th nucleotide from the 5′ end of each miRNA. These results confirmed that the genes chosen in this study were the specific targets of the corresponding miRNAs (Figure 4).

**Figure 5:**
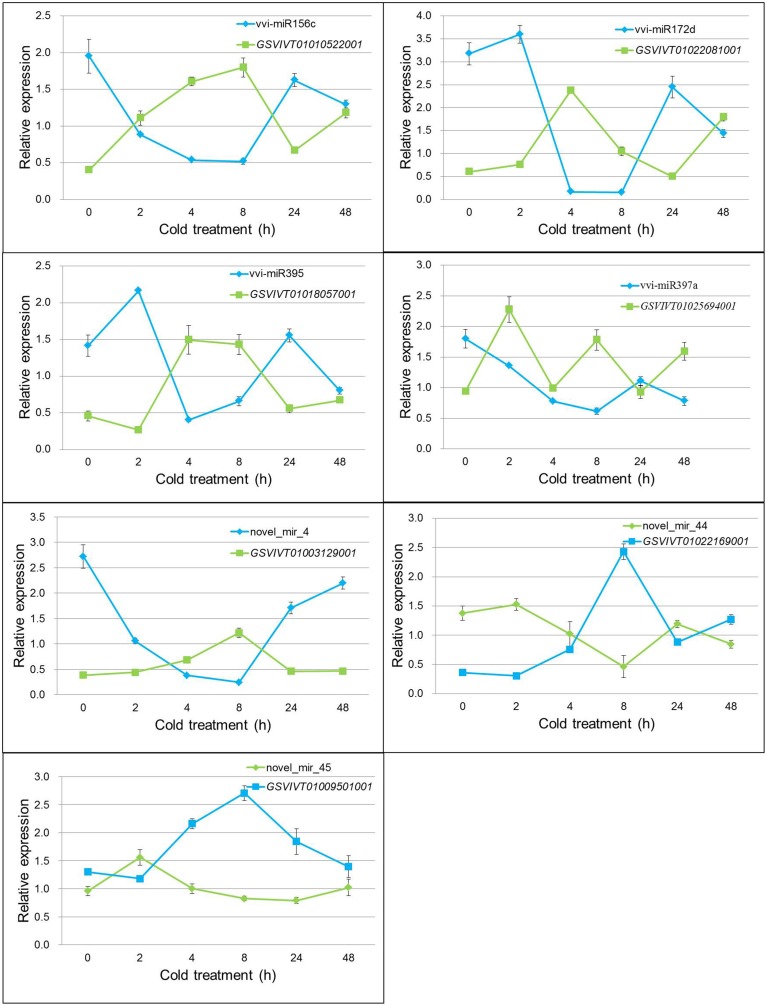
**Expression pattern correlation between vvi-miRNAs and their target genes**. Total RNA was extracted from grape leaves from micropropagated plantlets subjected to low temperature at 4°C for 0, 2, 4, 8, 24, and 48 h. The level of expression was normalized to that of the internal control. Error bars represent the standard deviations of three PCR replicates of a single reverse transcription reaction.

The transcription levels of the seven target genes under cold treatment in grapevine were detected by real-time RT-PCR (Figure 5). All genes showed increased expression patterns during cold stress, but reached their peak values at different cold-treated times. Negative correlation was found when the relative expression values from miRNAs and their relevant targets were combined. Pearson correlation coefficient analysis showed that there was a strong and significant negative correlation between the expression level of miRNAs and their target genes, such as vvi-miR156c and *GSVIVT01010522001* (Pearson correlation *r* = −0.959, *P* < 0.01), novel_mir_44 and *GSVIVT01022169001* (Pearson correlation *r* = −0.958, *P* < 0.01) (Table S10). The other five miRNAs and their targets also showed significant negative correlation in different degrees (Table S10). The previous results imply that the dependent transcription regulation of miRNAs was conducted through cleavage mode.

## Discussion

In recent years, increasing evidences suggest that miRNAs play critical roles in both biotic and abiotic stress responses. However, a systematic identification of cold-responsive miRNAs from *V. vinifera* had not been reported so far. Taking advantage of the sequenced genome of *V. vinifera* cv. Pinot Noir (PN40024) and the high-throughput sequencing technology, the present study reports the first identification of cold-inducible miRNAs in *V. vinifera*. We constructed two small RNA libraries and identified 163 known miRNAs and 67 novel miRNAs (Table S4 and S6). The 163 known miRNAs belonged to 22 conserved miRNA families and 24 non-conserved miRNA families. Previous studies indicate that 22 conserved miRNA families in both monocot and dicot model species exist (Jones-Rhoades et al., 2006; Rajagopalan et al., 2006). All conserved miRNA families were detected in these two libraries. The 67 novel miRNAs significantly increased the number of miRNAs in *V. vinifera*. As expected, most of the known miRNAs identified in *V. vinifera* were highly conserved in diverse plant species (Sunkar and Jagadeeswaran, 2008; Sunkar et al., 2008).

Identification of cold-inducible miRNAs has been reported in several species, such as *Arabidopsis* (Sunkar and Zhu, 2004; Liu et al., 2008; Zhou et al., 2008), rice (Lv et al., 2010; Barrera-Figueroa et al., 2012), Brachypodium (Zhang et al., 2009), Poncirus (Zhang et al., 2014), wheat (Wang et al., 2014a), and populus (Chen et al., 2012). Conserved miRNAs identified as cold-inducible were mainly from seedings and leaves. Summarizing the existing research, 21 out of the 22 conserved miRNA families were identified as cold-responsive (Table S9). Significant differences were found among different species. In this study, the expression of six conserved miRNAs, including miR156, miR171, miR172, miR395, miR397, and miR398, were downregulated after cold stress, and there was no conserved miRNAs showed significant upregulated. While in *Arabidopsis* and Brachypodium, only upregulated miRNAs were reported. All the six conserved miRNAs identified in our study were also confirmed to respond to cold stress in other species, but its expression pattern was different from each other. Several conserved miRNAs, including miR169, miR319, miR393, and miR408, were reported to respond to cold stress in other species; however, no visible expression change was found in our experiment. One interpretation for these differentials is that the induction levels of these miRNAs are too low to be recognized as significant changes in the present experimental system. These miRNAs may also possibly respond to cold stress only in specific tissues or at specific growth and developmental stages in grapevine. With the exception of conserved miRNAs, many cold-responsive miRNAs are species-specific. A previously identified cold-responsive miRNA, miR402 in *Arabidopsis* (Sunkar and Zhu, 2004), was not detected in our two libraries nor found in other species, suggesting that it is probably an *Arabidopsis*-specific miRNA. In this study, five cold-inducible DEMs, miR3624, miR3633, miR3634, miR3636, and miR3640, were found in our libraries, but not reported in other species, indicating *Vitis*-specific property.

Given that miRNAs regulate gene expression post-transcriptionally by endonucleolytic cleavage or translational repression, target prediction is essential to gain insight into the regulatory functions of miRNAs. In this study, targets of 44 cold-responsive miRNAs were predicted (Table S8). The targets of conserved miRNAs, such as miR156, miR171, and miR172, had been investigated in several grapevine cultivars, and their functions were almost in accordance with the previous studies (Carra et al., 2009; Mica et al., 2010; Pantaleo et al., 2010; Wang et al., 2011a, 2012). For the 9 non-conserved and 10 novel vvi-miRNAs, our results revealed that the functions of most target genes are unknown. However, some vvi-miRNA could target genes that belong to MYB, bHLH, and bZIP transcription factors, which have been reported as important regulators that respond to cold stress (Agarwal et al., 2006; Liu et al., 2012; Huang et al., 2013). In addition, miRNAs of the same family may target the same genes with similar functions. For instance, vvi-156b, vvi-miR156c, and vvi-miR156d have the same 11 target genes that contain an SBP domain. A total of 15 out of 18 vvi-miR397a target genes are involved in copper ion-binding (Table S8). The functional analysis of these candidate target genes, combined with miRNA-mediated post-transcriptional regulation research, will finally help in the illustration of the roles of miRNAs under cold stress in grapevine.

Seven predicted target genes for the four known and three novel vvi-miRNAs showed specific cleavage sites corresponding to their miRNA complementary sequences. The cleavage sites were in accordance with the classic cleavage mode of miRNA-mediated degradation of target genes in several species (Wang et al., 2012; Zhang et al., 2013). The expression pattern of these seven genes also showed negative correlation with the expression levels of the corresponding vvi-miRNAs. For the target genes of the five downregulated vvi-miRNAs under cold stress, the transcripts of *GSVIVT01010522001, GSVIVT01022081001, GSVIVT01018057001* and *GSVIVT01025694001*, and *GSVIVT01003129001* increased after the plantlets were subjected to cold stress. These results suggest that the SBP domain protein, AP2 transcription factor, sulfate adenylyltransferase, multicopper oxidase and E1-E2 ATPase can be induced by low temperature and may play an important role in responding to cold stress. Previous studies revealed that many AP2 family transcription factors, such as CBF1, CBF2, CBF3, and CBF4, play a crucial role in the cold signal pathway (Chinnusamy et al., 2007; Xiao et al., 2008; Li et al., 2013; Zhang et al., 2013). However, the functions of the SBP domain protein, sulfate adenylyltransferase, multicopper oxidase and E1-E2 ATPase under cold stress are still unknown. Combined with the miRNA regulation mechanism, finding the concrete functions of these proteins under various environment stresses is interesting.

## Conclusion

We identified 163 known miRNAs and 67 novel miRNAs; among which, 44 were differentially expressed miRNAs (DEMs) during cold stress in *V. vinifera*. The prediction of the target genes of DEMs indicates that miRNA may regulate some transcription factors, including AP2, SBP, MYB, bHLH, GRAS, and bZIP during cold stress. The expression patterns of 13 DEMs were verified by real-time RT-PCR analysis. Seven predicted target genes for four known and three novel vvi-miRNAs showed specific cleavage sites at around the 10th nucleotide from the 5′ end of the miRNA corresponding to their miRNA complementary sequences. The expression pattern of the predicted target genes revealed negative correlation with the expression level of corresponding vvi-miRNAs. The present study provides useful information for further investigations on the roles of cold-inducible miRNAs.

## Author contributions

HX, SL, and XS designed and oversaw the research. XS, GF, and LS performed the experiments and took care of the plants. WW and ZL participated in the sequence analysis and helped to modify the manuscript. XS and HX analyzed the data. XS, HX, and SL wrote the manuscript, made the figures and finalized the tables. All authors read and approved the final manuscript.

### Conflict of interest statement

The authors declare that the research was conducted in the absence of any commercial or financial relationships that could be construed as a potential conflict of interest.

## Supplementary material

Figure S1 **Secondary structures of the 67 putative novel miRNAs**.

Figure S2 **Real-time RT-PCR validations of two non-differentially expressed vvi-miRNAs**.

Table S1 **Raw reads generated by small RNA sequencing**.

Table S2 **Mapping statistic of the non-cold-treated (NCT) condition and subjected to cold stress (CT) at 4°C libraries**.

Table S3 **Distribution of different small RNAs categories in non-cold-treated (NCT) condition and subjected to cold stress (CT) at 4° libraries**.

Table S4 **Detail information of known miRNAs identified in *V. vinifera***.

Table S5 **Detailed information of sequence-variants for known miRNAs**.

Table S6 **Novel miRNAs identified in *V. vinifera***.

Table S7 **Identification of differently expressed miRNAs during cold treatment in *V. vinifera***.

Table S8 **Functional annotations for the predicted target genes of the cold-inducible vvi-miRNAs**.

Table S9 **List of cold-responsive conserved miRNAs identified in different species**.

Table S10 **The expression correlation between miRNA and its target gene**.

Table S11 **Primer sequences designed in this study**.
