# Supplementary material for: Identification of cold-inducible microRNAs in grapevine
Source: Front Plant Sci. 2015 Aug 4;6:595. doi: 10.3389/fpls.2015.00595 (PMC4523783; doi:10.3389/fpls.2015.00595)
Supplement: Figure S1 — Secondary structures of the 67 putative novel miRNAs. [file Image1.PDF]

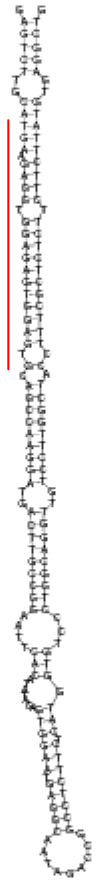

novel_mir_1

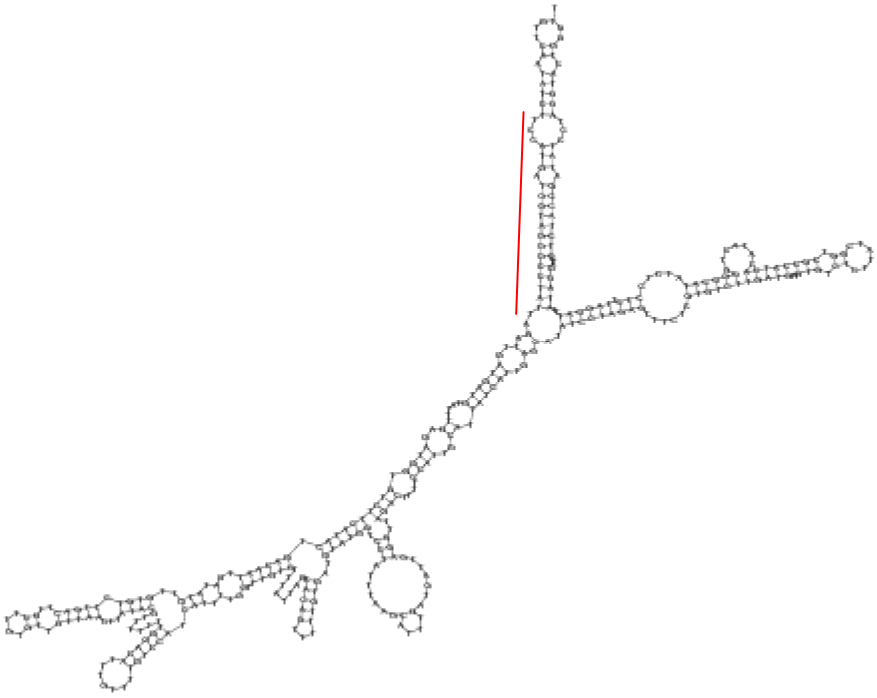

novel_mir_2

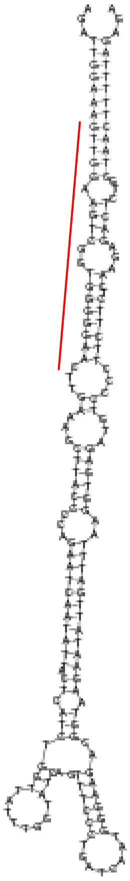

novel_mir_3

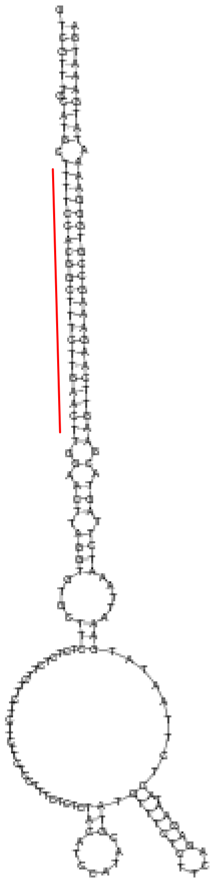

novel_mir_4

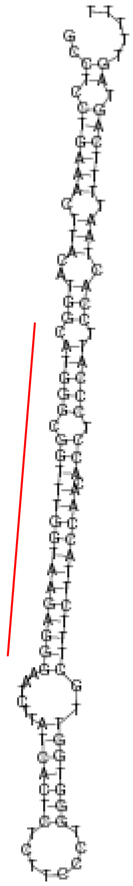

novel_mir_5

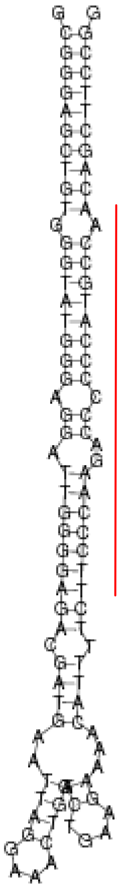

novel_mir_6

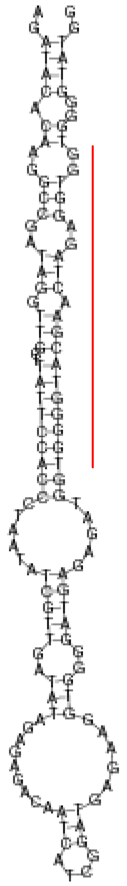

novel_mir_7

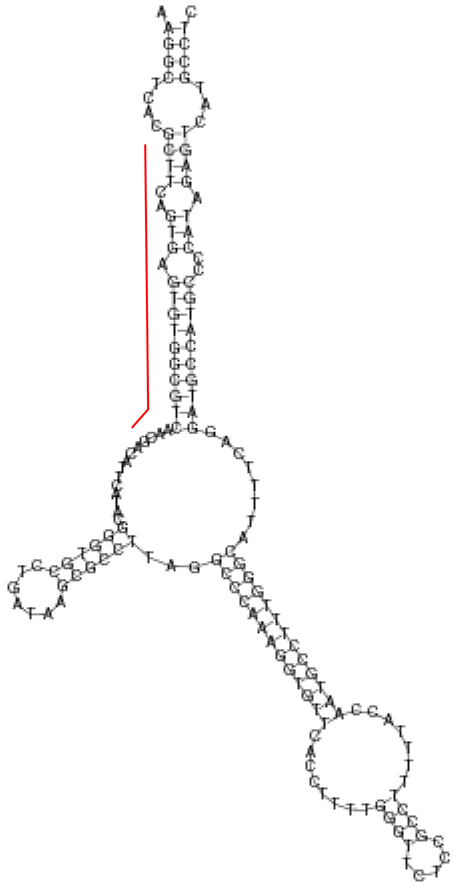

novel_mir_8

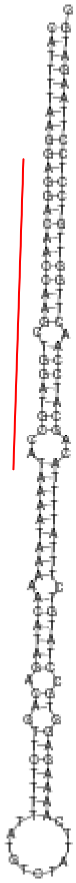

novel_mir_9

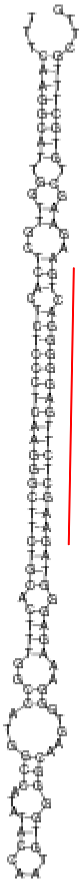

novel_mir_10

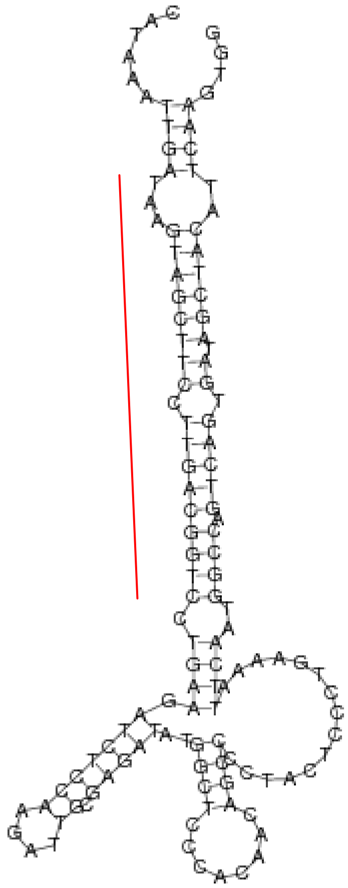

novel_mir_11

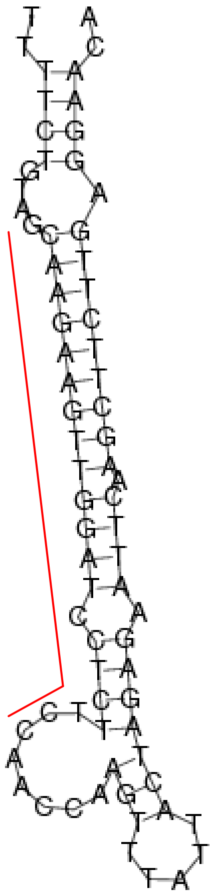

novel_mir_12

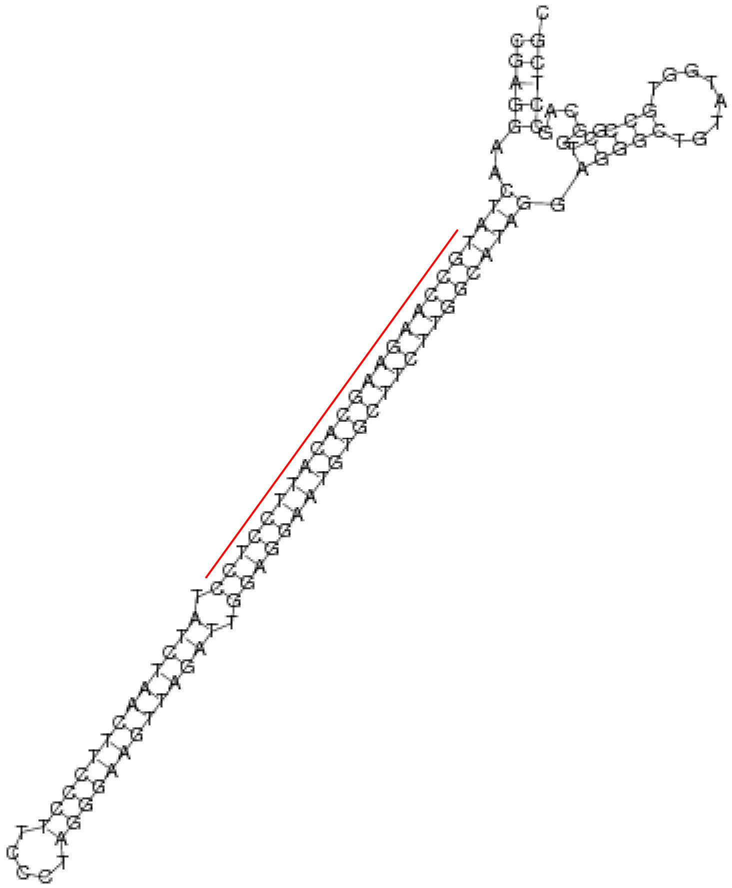

novel_mir_13

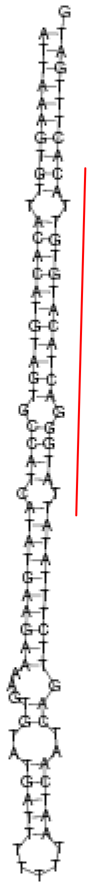

novel_mir_14

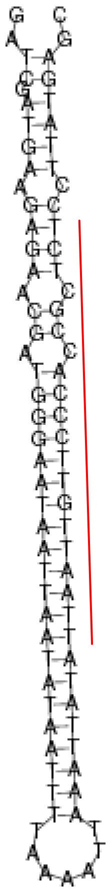

novel_mir_15

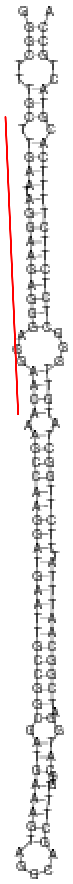

novel_mir_16

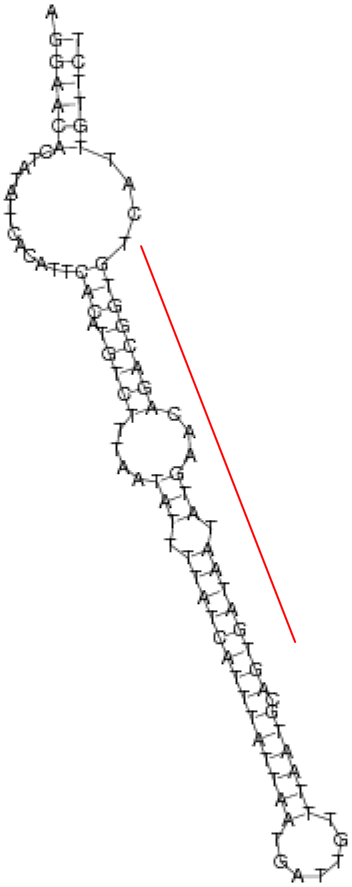

novel_mir_17

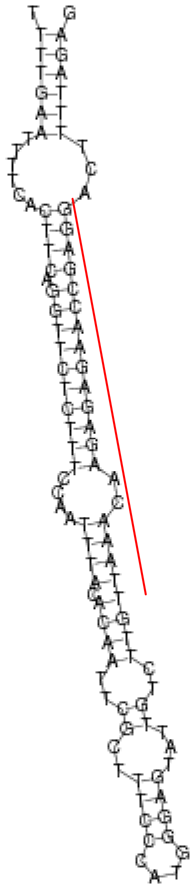

novel_mir_18

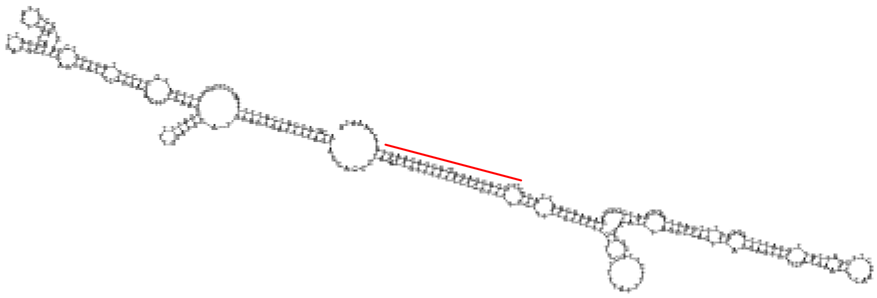

novel_mir_19

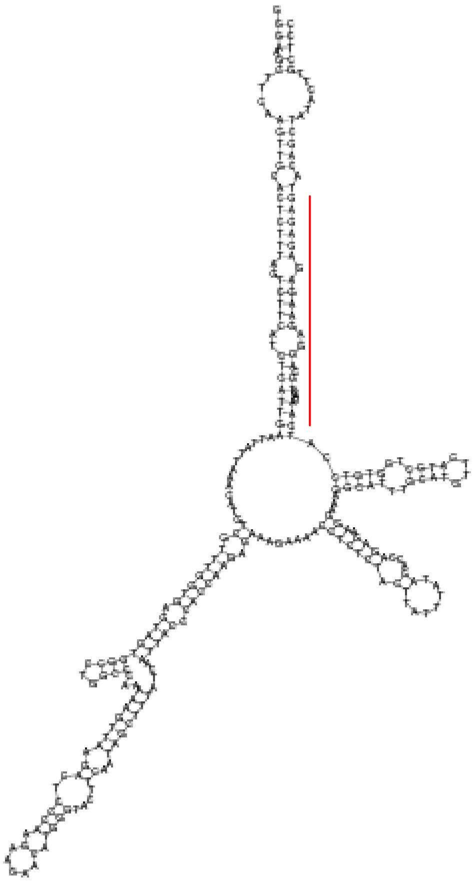

novel_mir_20

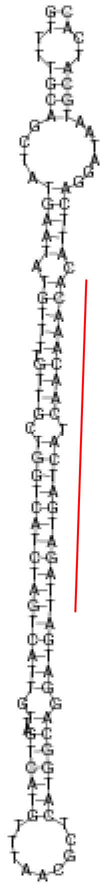

novel_mir_21

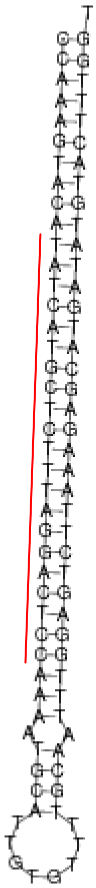

novel_mir_22

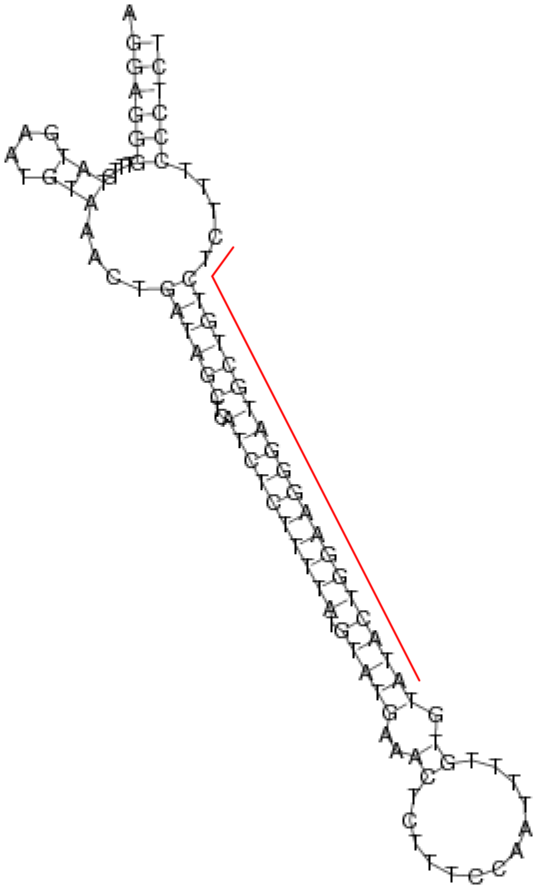

novel_mir_23

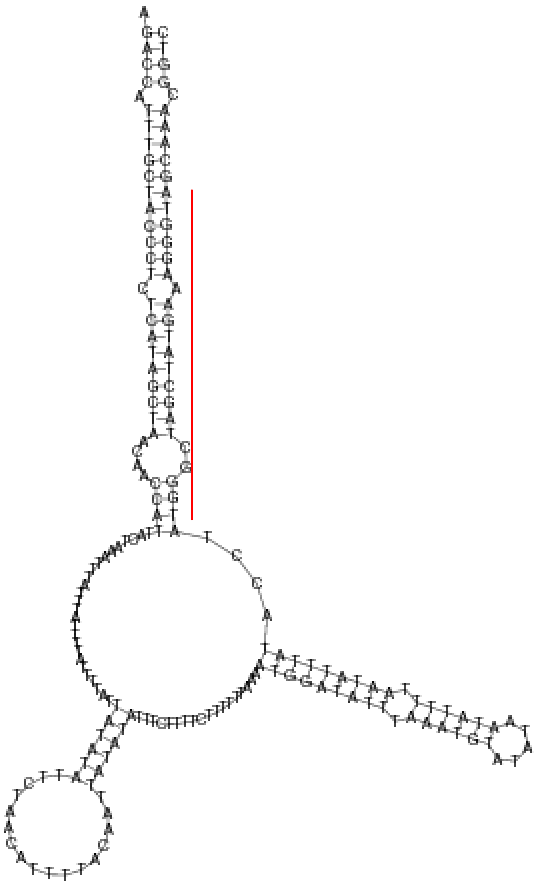

novel_mir_24

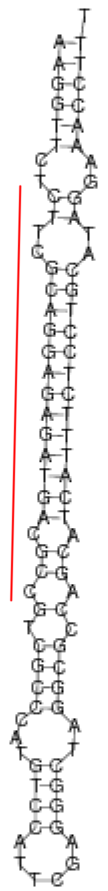

novel_mir_25

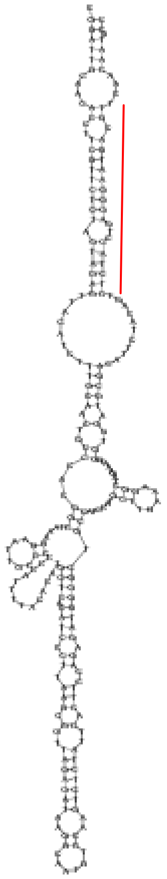

novel_mir_26

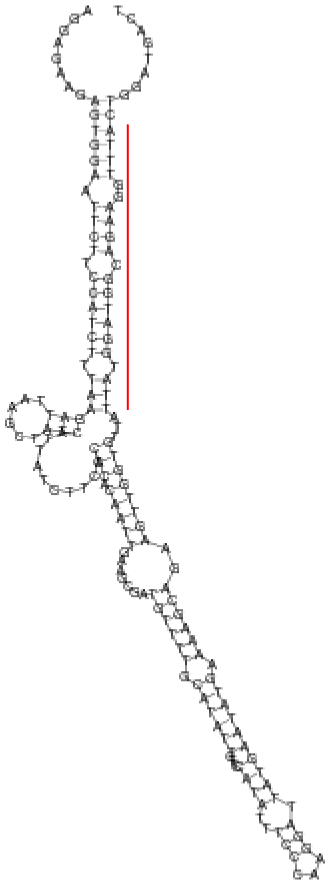

novel_mir_27

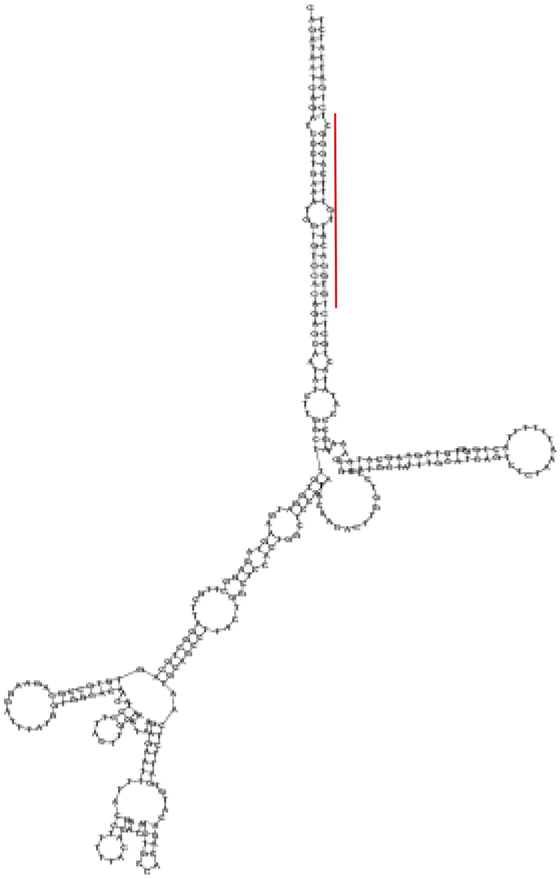

novel_mir_28

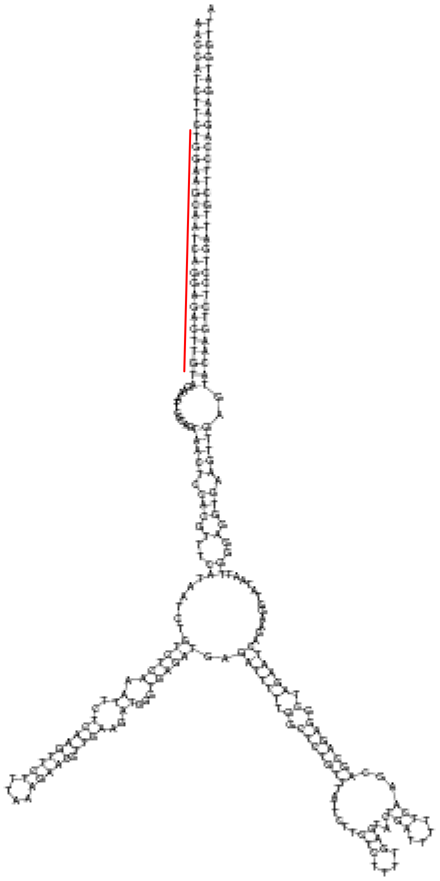

novel_mir_29

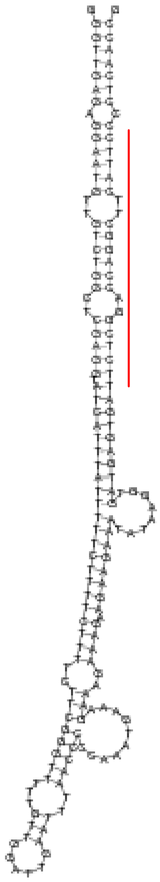

novel_mir_30

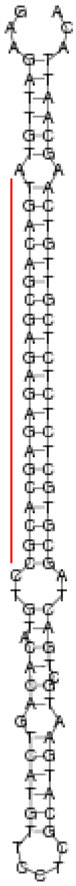

novel_mir_31

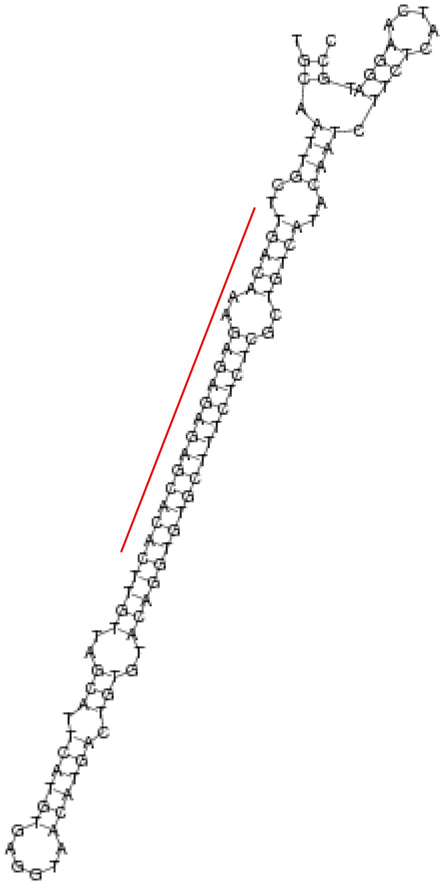

novel_mir_32

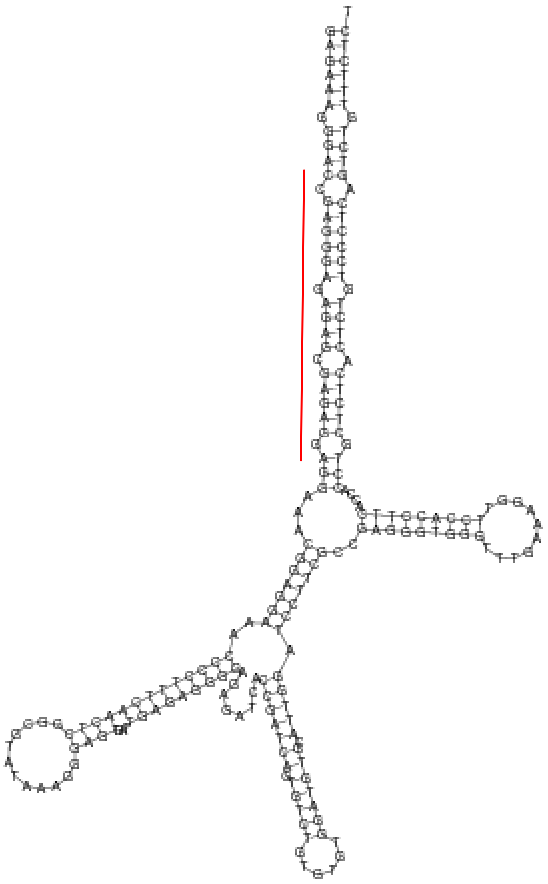

novel_mir_33

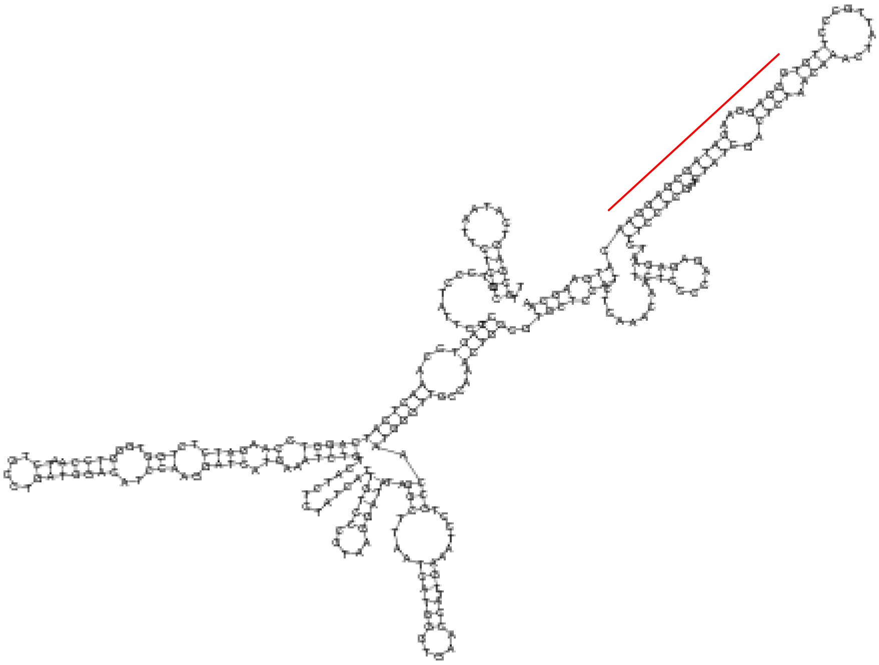

novel_mir_34

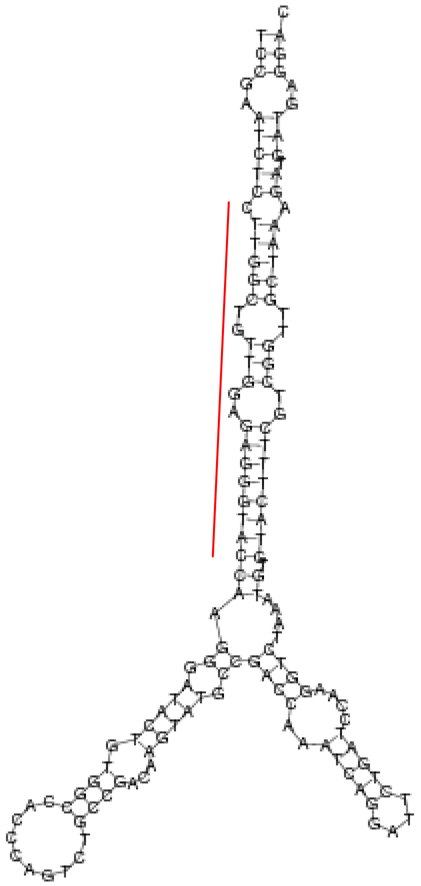

novel_mir_35

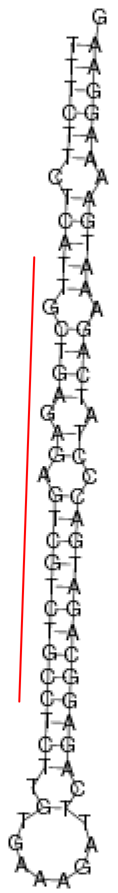

novel_mir_36

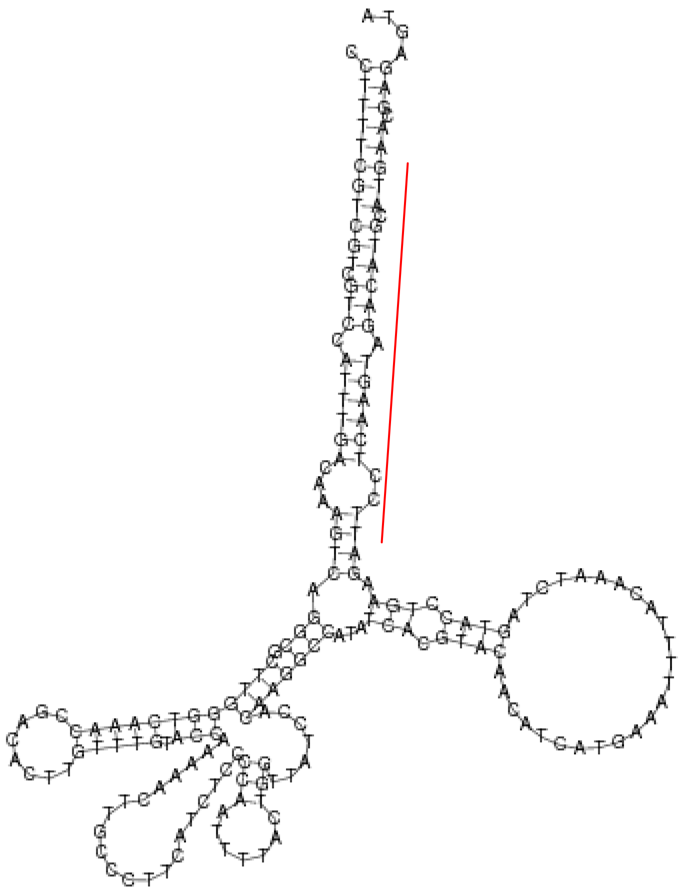

novel_mir_37

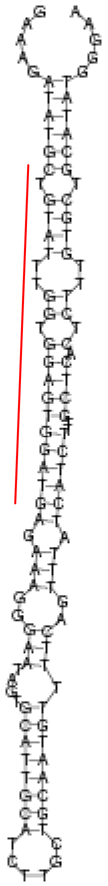

novel_mir_38

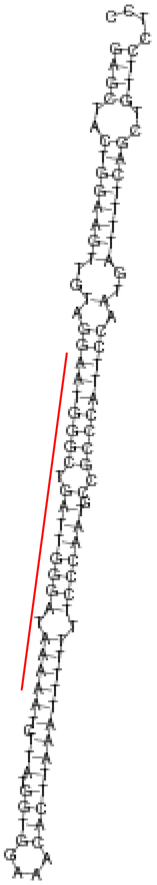

novel_mir_39

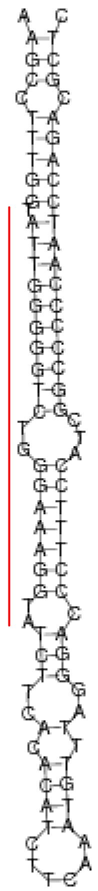

novel_mir_40

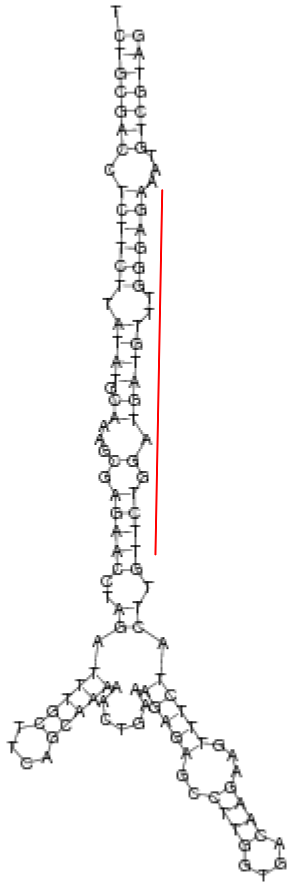

novel_mir_41

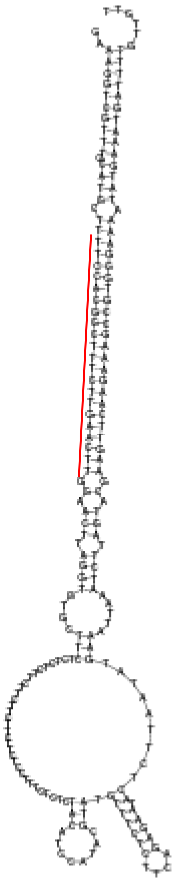

novel_mir_42

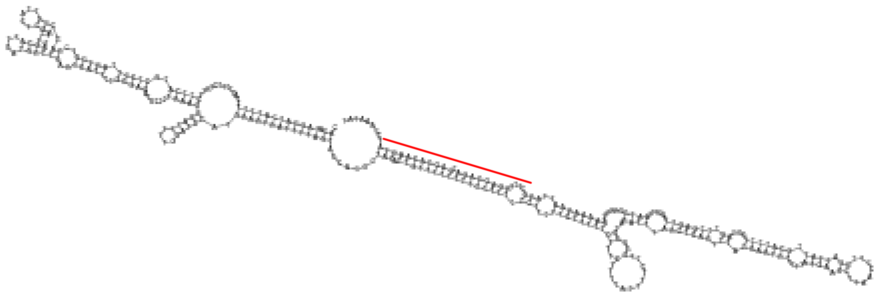

novel_mir_43

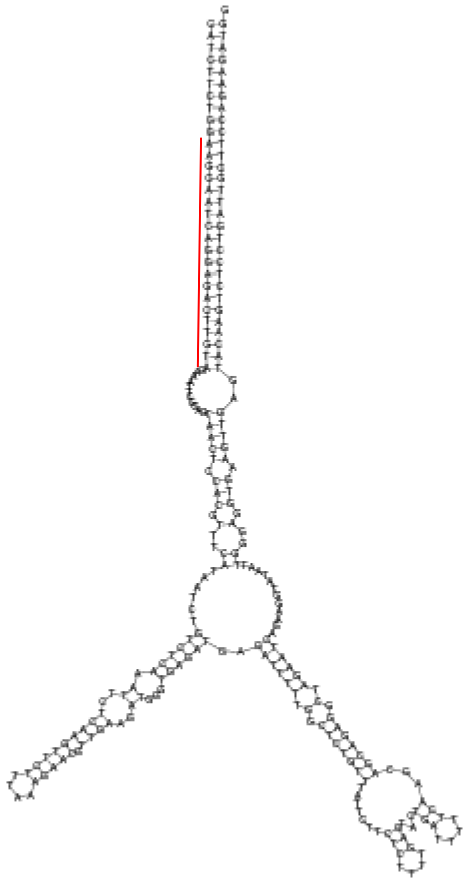

novel_mir_44

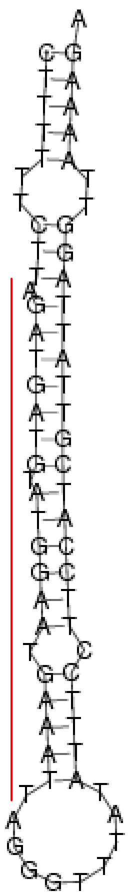

novel_mir_45

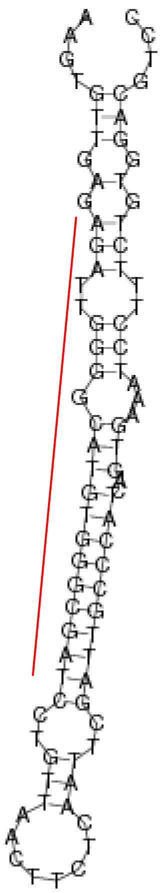

novel_mir_46

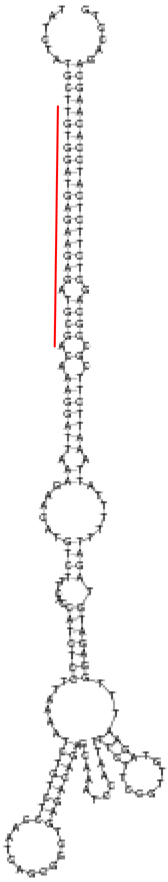

novel_mir_47

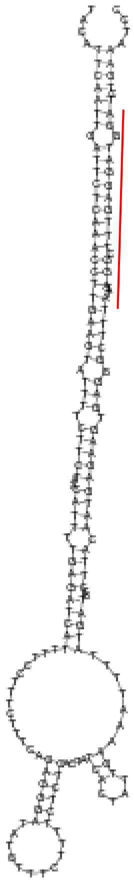

novel_mir_48

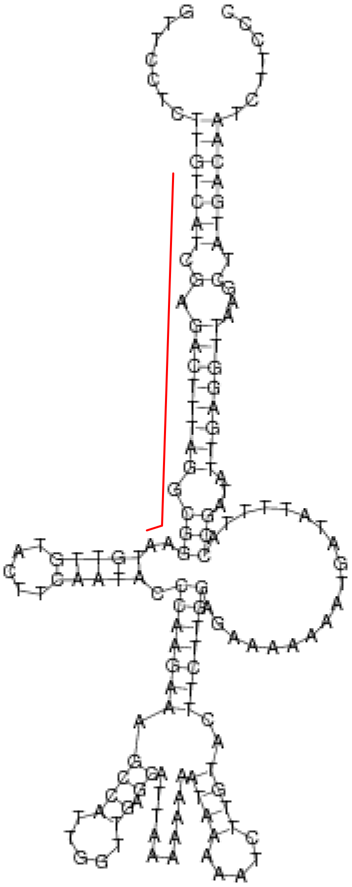

novel_mir_49

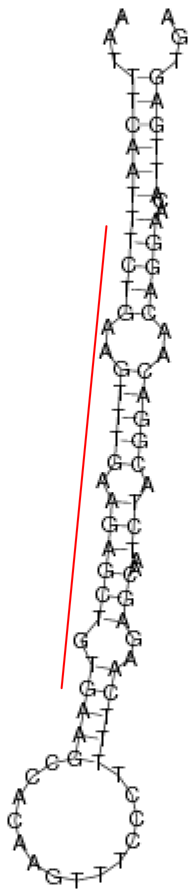

novel_mir_50

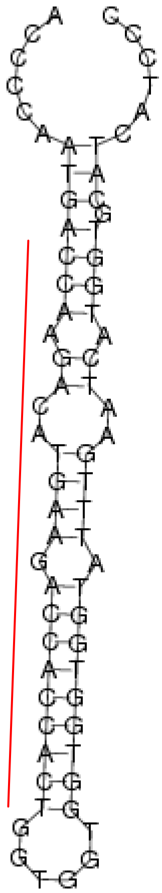

novel_mir_51

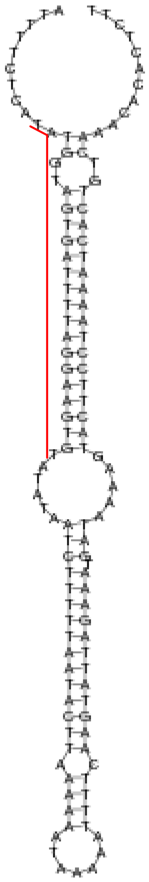

novel_mir_52

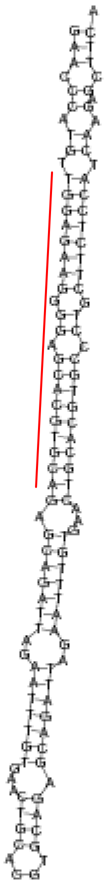

novel_mir_53

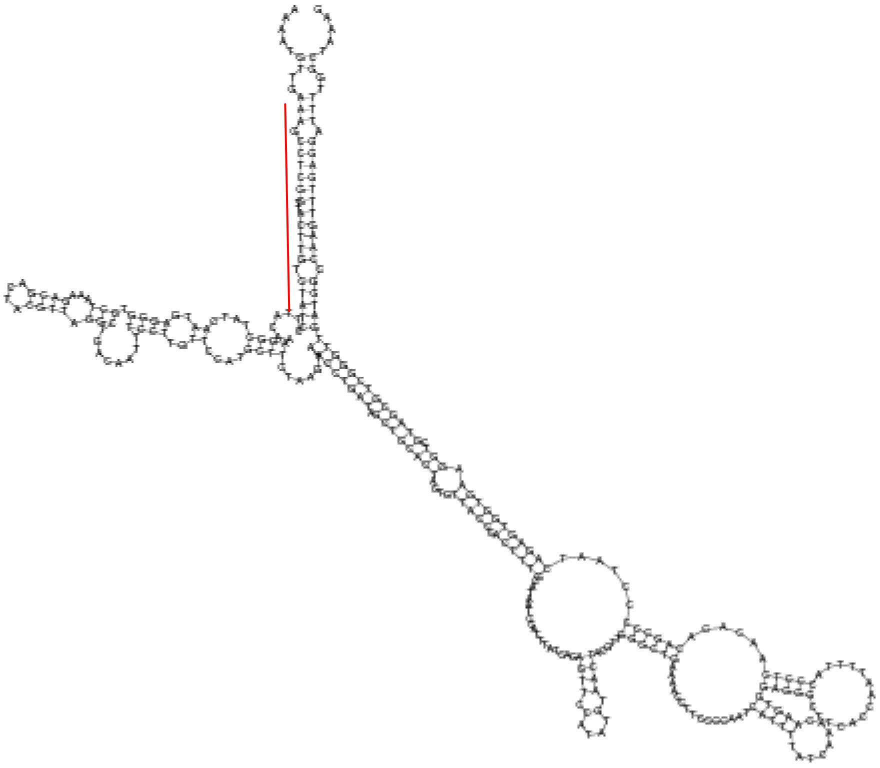

novel_mir_54

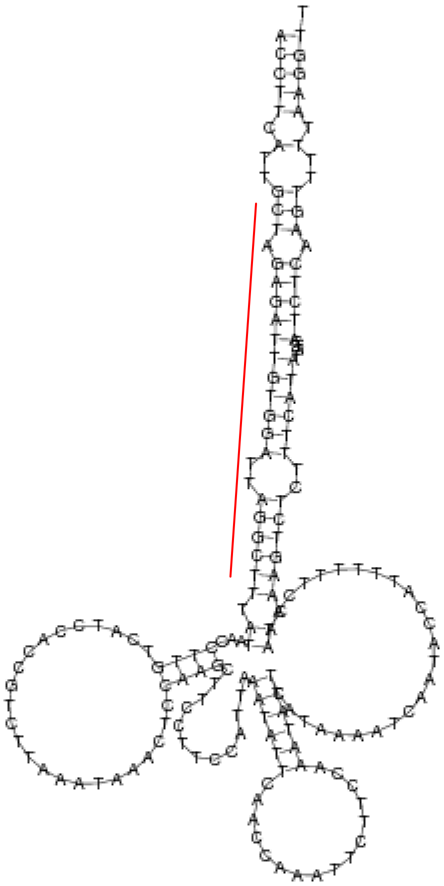

novel_mir_55

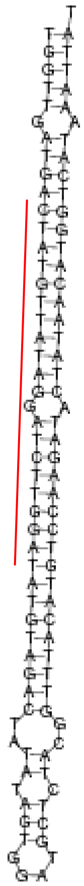

novel_mir_56

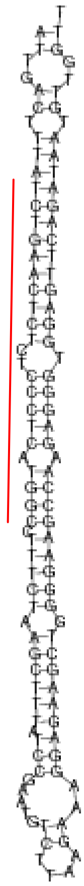

novel_mir_57

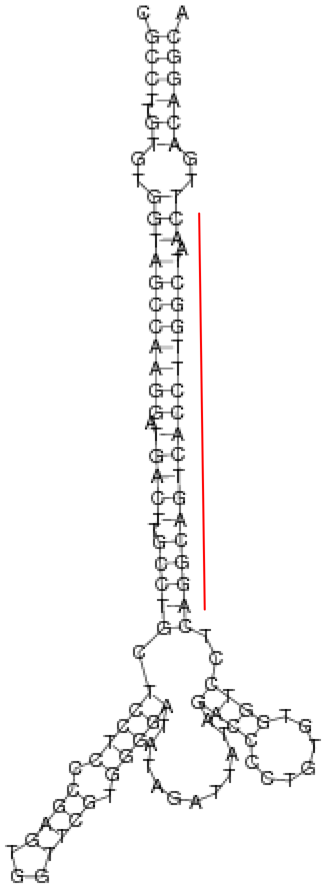

novel_mir_58

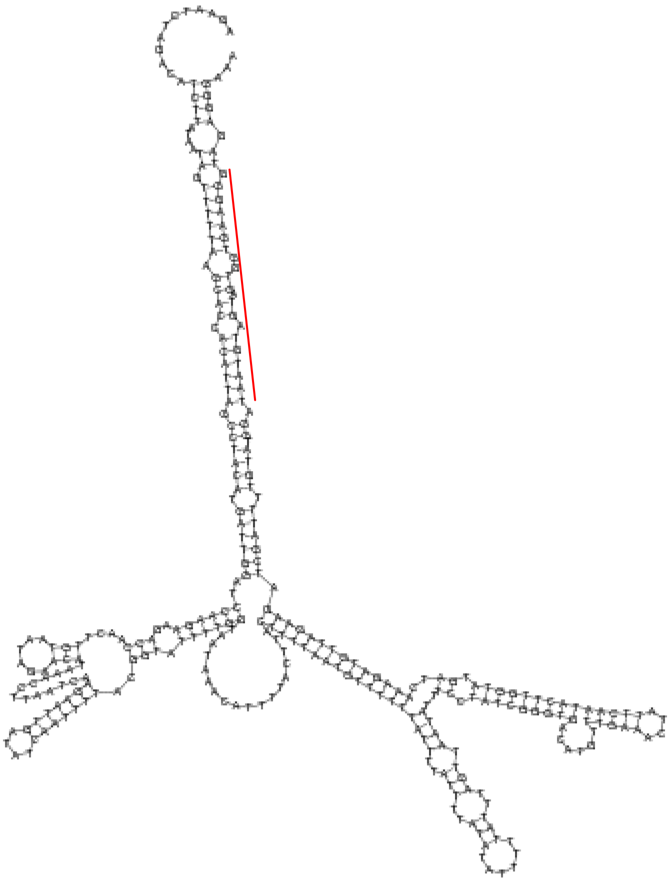

novel_mir_59

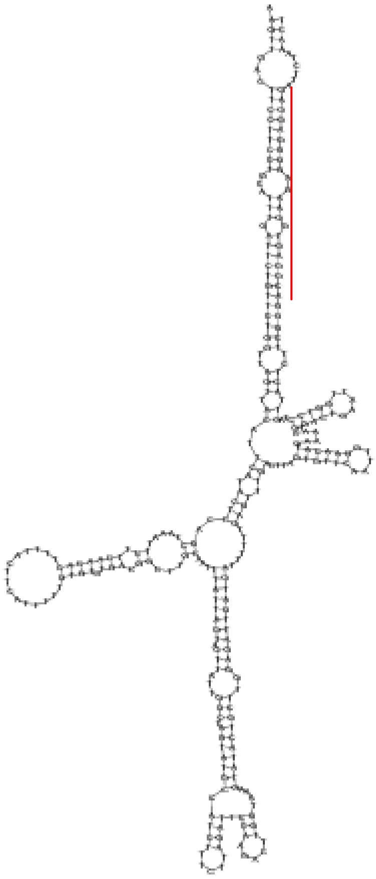

novel_mir_60

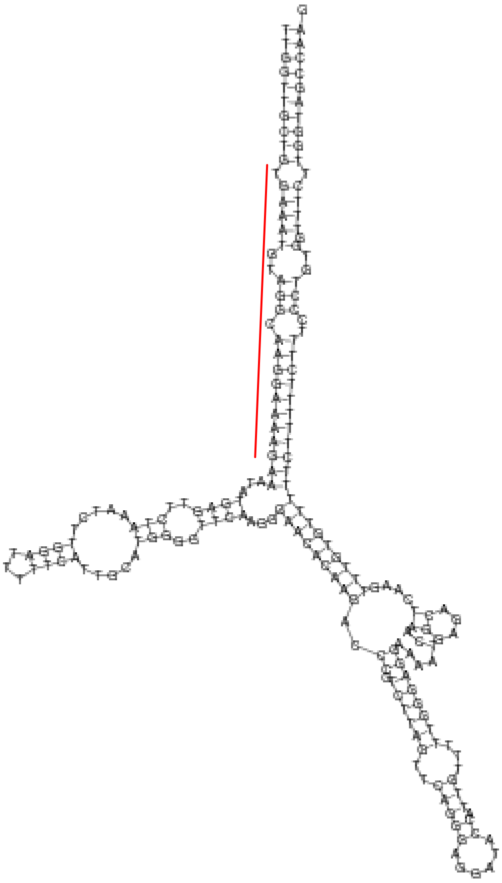

novel_mir_61

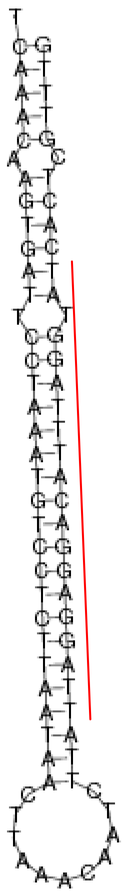

novel_mir_62

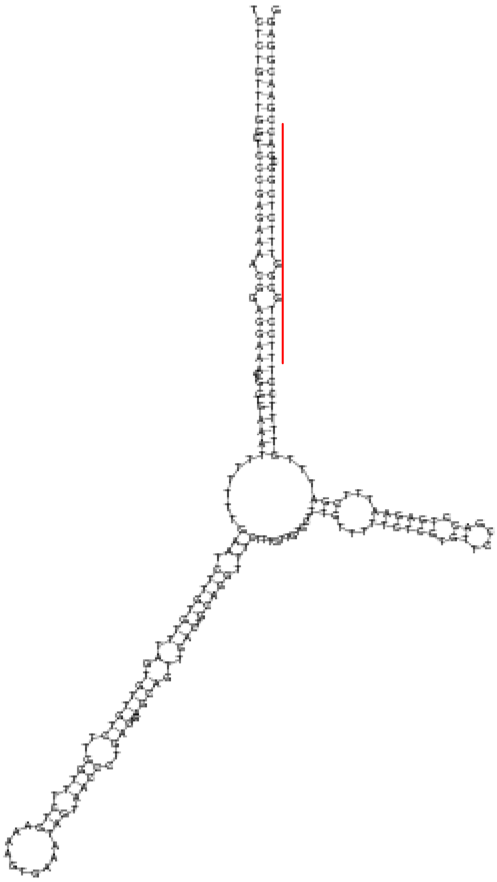

novel_mir_63

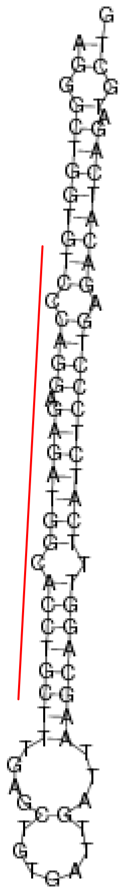

novel_mir_64

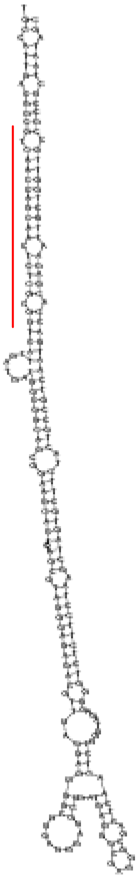

novel_mir_65

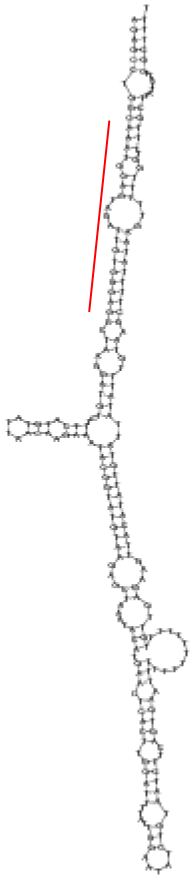

novel_mir_66

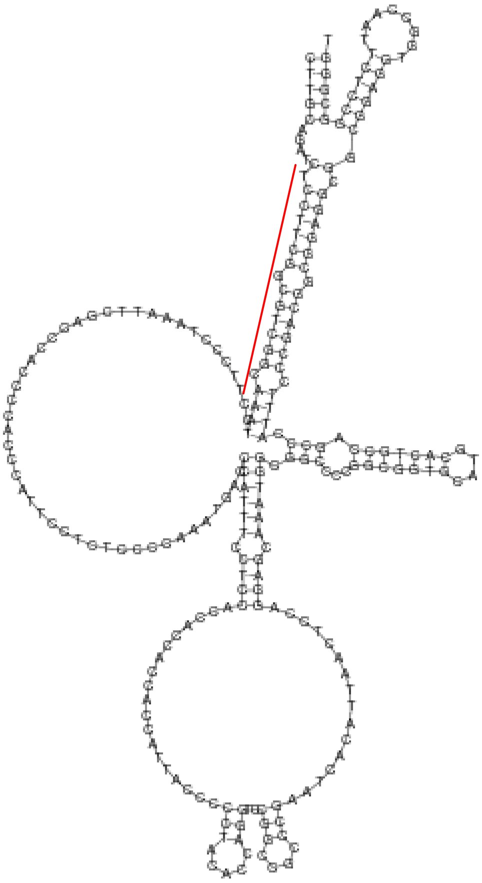

novel_mir_67
