# Supplementary figures and images for: Identification of cold-inducible microRNAs in grapevine
Source: Front Plant Sci. 2015 Aug 4;6:595. doi: 10.3389/fpls.2015.00595 (PMC4523783; doi:10.3389/fpls.2015.00595)

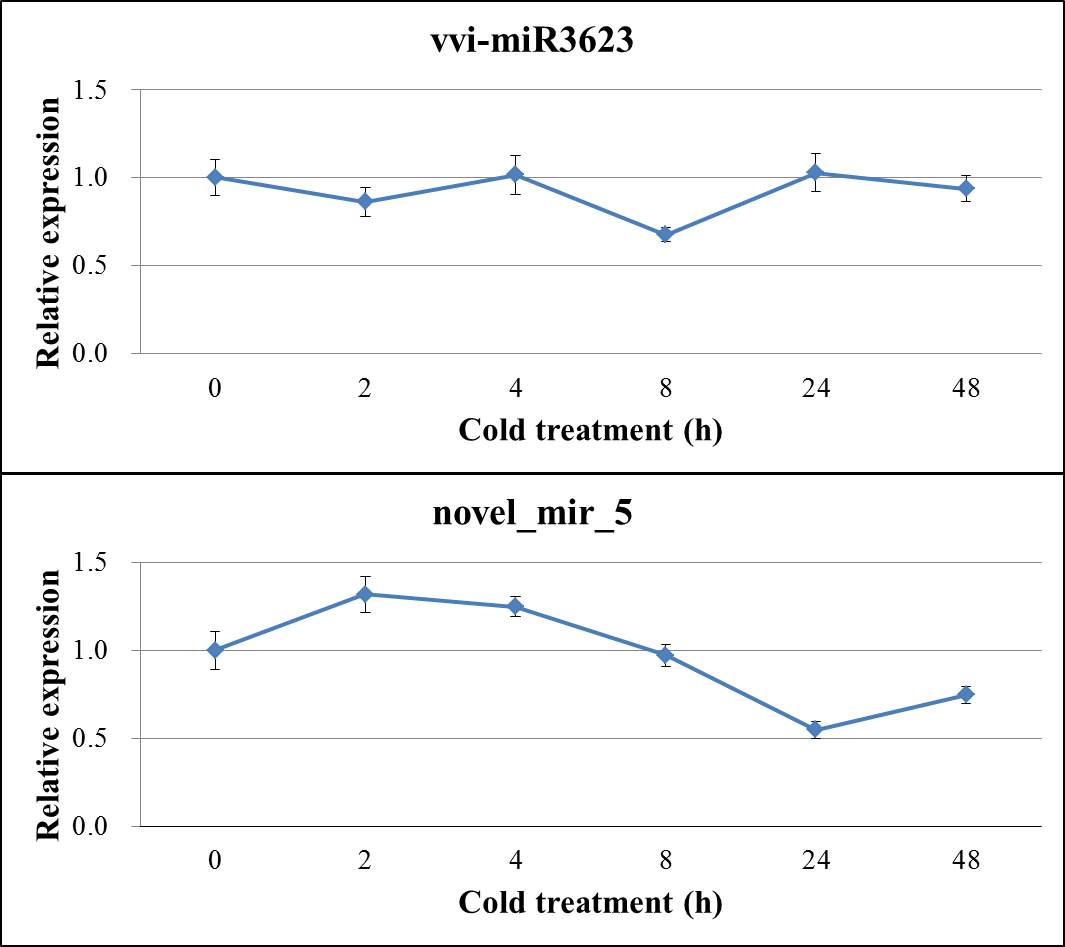

Supplement: Figure S2 — Real-time RT-PCR validations of two non-differentially expressed vvi-miRNAs. [file Image2.JPEG]
