# Supplementary material for: Identification of cold-inducible microRNAs in grapevine
Source: Front Plant Sci. 2015 Aug 4;6:595. doi: 10.3389/fpls.2015.00595 (PMC4523783; doi:10.3389/fpls.2015.00595)
Supplement: Table S1 — Raw reads generated by small RNA sequencing. [file Table1.DOCX]

**Table S1 Raw reads generated by small RNA sequencing.**

| **Type** | **NCT^a^** | | **CT^b^** | |
| --- | --- | --- | --- | --- |
|  | **Count** | **Percent (%)** | **Count** | **Percent (%)** |
| Raw reads | 21749505 | 100% | 26320458 | 100% |
| 3'adapter null | 12472 | 0.06% | 19094 | 0.07% |
| Insert null | 6731 | 0.03% | 5936 | 0.02% |
| 5'adapter contaminants | 155155 | 0.71% | 362096 | 1.38% |
| Smaller than 18nt | 218975 | 1.01% | 16120 | 0.06% |
| polyA | 772 | 0.00% | 1397 | 0.01% |
| Clean reads | 21355400 | 98.19% | 25915815 | 98.46% |

^a^ NCT means small RNA library generated from non-cold-treated sample.

^b^ CT means small RNA library generated from cold-treated sample.
