# Supplementary material for: Identification of cold-inducible microRNAs in grapevine
Source: Front Plant Sci. 2015 Aug 4;6:595. doi: 10.3389/fpls.2015.00595 (PMC4523783; doi:10.3389/fpls.2015.00595)
Supplement: Table S2 — Mapping statistic of the non-cold-treated (NCT) condition and subjected to cold stress (CT) at 4°C libraries. [file Table2.DOCX]

**Table S2 Mapping statistic of the non-cold-treated (NCT) condition and subjected to cold stress (CT) at 4 ^o^C libraries.**

| **Category** | **NCT Library** | **CT Library** |
| --- | --- | --- |
| **Total sRNAs** | 21355400 | 25915815 |
| **Mapping to genome** | 15460249 | 18443742 |
| **Percent (%)** | 72.40% | 71.17% |
| **Unique sRNAs** | 2633523 | 4459318 |
| **Mapping to genome (%)** | 1565788 | 2565772 |
| **Percent (%)** | 59.46% | 57.54% |
