# Supplementary material for: Identification of cold-inducible microRNAs in grapevine
Source: Front Plant Sci. 2015 Aug 4;6:595. doi: 10.3389/fpls.2015.00595 (PMC4523783; doi:10.3389/fpls.2015.00595)
Supplement: Table S3 — Distribution of different small RNAs categories in non-cold-treated (NCT) condition and subjected to cold stress (CT) at 4° libraries. [file Table3.DOCX]

**Table S3. Distribution of different small RNAs categories in non-cold-treated (NCT) condition and subjected to cold stress (CT) at 4 oC libraries.**

| **Category** | **NCT Library** | | | | **CT Library** | | | |
| --- | --- | --- | --- | --- | --- | --- | --- | --- |
|  | **Unique sRNAs** | **Percent (%)** | **Total sRNAs** | **Percent (%)** | **Unique sRNAs** | **Percent (%)** | **Total sRNAs** | **Percent (%)** |
| **Total** | 2633523 | 100% | 21355400 | 100% | 4459318 | 100% | 25915815 | 100% |
| **exon_antisense** | 60936 | 2.31% | 254657 | 1.19% | 75949 | 1.70% | 294013 | 1.13% |
| **exon_sense** | 169379 | 6.43% | 764059 | 3.58% | 174139 | 3.91% | 782731 | 3.02% |
| **intron_antisense** | 48940 | 1.86% | 135816 | 0.64% | 85349 | 1.91% | 254492 | 0.98% |
| **intron_sense** | 75102 | 2.85% | 398166 | 1.86% | 118990 | 2.67% | 705396 | 2.72% |
| **miRNA** | 1431 | 0.05% | 6404541 | 29.99% | 1479 | 0.03% | 6148881 | 23.73% |
| **rRNA** | 119270 | 4.53% | 3212579 | 15.04% | 138718 | 3.11% | 2612687 | 10.08% |
| **repeat** | 496028 | 18.84% | 1520951 | 7.12% | 888058 | 19.91% | 3621485 | 13.97% |
| **snRNA** | 2385 | 0.09% | 8860 | 0.04% | 2868 | 0.06% | 8564 | 0.03% |
| **snoRNA** | 1948 | 0.07% | 13330 | 0.06% | 1919 | 0.04% | 8359 | 0.03% |
| **tRNA** | 16645 | 0.63% | 1184489 | 5.55% | 16271 | 0.36% | 626235 | 2.42% |
| **unannotated** | 1641459 | 62.33% | 7457952 | 34.92% | 2955578 | 66.28% | 10852972 | 41.88% |
